# Technologies, Clinical Applications, and Implementation Barriers of Digital Twins in Precision Cardiology: Systematic Review

**DOI:** 10.2196/78499

**Published:** 2026-01-08

**Authors:** Fatemeh Sarani Rad, Ehsan Bitaraf, Maryam Jafarpour, Juan Li

**Affiliations:** 1 Computer Science Department, North Dakota State University, 1320 Albrecht Blvd, Fargo, ND, 58105, United States, 1 (701) 231-9662; 2 Center for Technology and Innovation in Cardiovascular Informatics, Rajaie Cardiovascular Medical and Research Center, Iran University of Medical Sciences, Tehran, Iran; 3 Center for Medical Data Science, Medical University of Vienna, Vienna, Austria

**Keywords:** digital twin, cardiology, personalized medicine, simulation, machine learning, clinical decision support, ethics

## Abstract

**Background:**

Digital twin systems are emerging as promising tools in precision cardiology, enabling dynamic, patient-specific simulations to support diagnosis, risk assessment, and treatment planning. However, the current landscape of cardiovascular digital twin development, validation, and implementation remains fragmented, with substantial variability in modeling approaches, data use, and reporting practices.

**Objective:**

This systematic review aims to synthesize the current state of cardiovascular digital twin research by addressing 11 research questions spanning modeling technologies, data infrastructure, clinical applications, clinical impact, implementation barriers, and ethical considerations.

**Methods:**

We systematically searched 5 databases (PubMed, Scopus, Web of Science, IEEE Xplore, and Google Scholar) and screened 330 records. Forty-two original studies met the predefined eligibility criteria and were included. Data extraction was guided by 11 thematic research questions. Mechanistic and artificial intelligence (AI) or machine learning (ML) modeling strategies, data modalities, visualization formats, clinical use cases, reported impacts, limitations, and ethical or legal issues were coded and summarized. Risk of bias was evaluated using a custom checklist for modeling studies, the Prediction Model Risk of Bias Assessment Tool (PROBAST) for prediction models, and the Risk of Bias in Non-Randomized Studies - of Interventions (ROBINS-I) for observational studies.

**Results:**

Most digital twins (29/42, 69%) relied on mechanistic models, while hybrid mechanistic–data-driven approaches and purely data-driven designs were less frequent (13/42, 31%). Only 18 studies explicitly described ML or AI, most often deep learning, Bayesian methods, or optimization algorithms. Personalization depended primarily on imaging (32/42, 76%) and electrocardiography or other electrical signals (18/42, 43%). Visualization was dominated (41/42, 98%) by static figures and anatomical snapshots. Clinically, digital twins were most commonly applied to therapy planning, risk prediction, and monitoring. Reported benefits focused on improved decision-making and therapy-related impacts, with occasional (8/42, 19%) reports of increased accuracy or faster diagnosis, but there was limited evidence for downstream improvements in patient outcomes. Key barriers included strong model assumptions and simplifications; high computational cost; data quality and availability constraints; limited external validation; and challenges in real-time performance, workflow integration, and usability. Explicit discussion of ethical, legal, or governance issues was rare (7/42, 17%).

**Conclusions:**

Cardiovascular digital twins show substantial potential to advance precision cardiology by linking personalized physiological models with clinical decision support, particularly for therapy planning and risk prediction in arrhythmia and heart failure. However, real-world implementation is constrained by methodological heterogeneity, restricted data and validation practices, limited openness of code and models, and sparse engagement with ethical and governance questions. Future research should prioritize standardized evaluation frameworks, robust clinical validation, interoperable and user-centered system design, and ethically grounded, patient-centered development to realize the full clinical value of digital twin systems.

## Introduction

In recent years, the integration of digital twin technology into health care has opened new avenues for precision medicine, particularly within the field of cardiology. A digital twin is a dynamic, virtual representation of a physical system that is continuously updated with real-time data, advanced computational models, and artificial intelligence (AI) analytics [1,2]. In the context of health care, digital twins serve as virtual replicas of patients, organs, or biological systems, encompassing multidimensional, patient-specific information to inform clinical decisions [3-5].

Cardiovascular diseases (CVDs) remain a leading cause of morbidity and mortality worldwide, underscoring the need for innovative, patient-centric approaches to diagnosis, treatment, and management [6,7]. The application of digital twins in cardiology involves the creation of virtual replicas of the human heart by integrating anatomical, physiological, and molecular data. These models are capable of simulating electrical activity [8], mechanical function, hemodynamics, and drug responses [9,10]. By combining data from cardiac imaging (eg, magnetic resonance imaging [MRI] and computed tomography [CT]), electrocardiography (ECG), hemodynamic profiles, electrophysiology recordings, electronic health records, and omics assessments, digital twin systems provide a basis for precision simulation and virtual experimentation [11].

These capabilities make cardiac digital twins uniquely positioned to support personalized treatment plans, enabling applications such as risk stratification, therapy optimization, surgical simulation, and drug safety testing. The integration of AI, particularly machine learning (ML) and deep learning (DL), has further improved the scalability and performance of digital twins in real-world applications.

However, despite promising technical progress, substantial challenges remain. These include (1) high computational costs and complex personalization pipelines; (2) data heterogeneity and interoperability limitations; (3) lack of standardized validation protocols and clinical benchmarks; and (4) ongoing concerns regarding privacy, explainability, and regulatory oversight.

While multiple reviews have surveyed digital twins in general health care [12] or addressed cardiovascular simulation from a technical standpoint [11], a comprehensive, domain-specific synthesis integrating technical, clinical, and implementation perspectives in personalized cardiology remains lacking.

To address this gap, we conducted a systematic review following the PRISMA (Preferred Reporting Items for Systematic Reviews and Meta-Analyses) 2020 guidelines [13,14]. This review explicitly examines original research articles on cardiovascular digital twin systems, emphasizing personalization, clinical relevance, and implementation feasibility. Our study followed a two-stage methodology:

1. Screening phase: We screened 330 articles from 5 databases (PubMed, Scopus, IEEE, Web of Science, and Google Scholar). After removing duplicates, non-English entries, and publications lacking abstracts or relevant context, 42 articles were retained.
2. Review phase: Three independent reviewers assessed full-text articles based on structured research questions (RQs). Each article was evaluated for relevance to 11 themes covering technology, data integration, clinical application, validation, ethics, and data sources.

The following RQs guided our review:

- RQ1-RQ4: What are the technological foundations of cardiovascular digital twins, including modeling strategies, AI integration, and open-source availability?
- RQ5 and RQ6: How is patient-specific data structured and visualized?
- RQ7 and RQ8: What are the clinical applications and disease targets of digital twins?
- RQ9: What clinical impacts have been reported as a result of digital twin use?
- RQ10 and RQ11: What barriers, limitations, and ethical or legal considerations are acknowledged in current studies?

The aim of this study was to systematically review the existing literature on cardiovascular digital twins to identify current technologies, clinical uses, and challenges to implementation.

## Methods

### Overview

This systematic review was designed and conducted following the PRISMA 2020 statement (Checklist 1). The protocol was developed in advance and used a transparent, reproducible approach to article retrieval, screening, and extraction. It was structured around 11 domain-specific RQs targeting the technological, clinical, and implementation dimensions of digital twin systems in cardiology.

### Data Sources and Search Strategy

A comprehensive literature search was performed across 5 major academic databases: PubMed, Scopus, Web of Science, IEEE Xplore, and Google Scholar. These platforms were selected to ensure broad interdisciplinary coverage across biomedical, engineering, and computational sciences. The databases were searched between January and early February 2025, restricting records to publications from 2010 onwards. Only the first 115 results sorted by relevance were screened for Google Scholar due to indexing limitations. The reference lists of relevant reviews were also scanned to ensure inclusion of key foundational articles.

Data collection and initial preprocessing were streamlined using Triple-A software [15], which served as the main tool for managing and organizing the retrieved records.

The search strategy used Boolean combinations of controlled vocabulary (eg, MeSH) and free-text terms as follows: (“digital twin” OR “virtual heart” OR “patient-specific model”) AND (“cardiology” OR “cardiac” OR “heart” OR “cardiovascular”) AND (“simulation” OR “personalized medicine” OR “precision medicine” OR “in silico”).

To increase transparency, we conceptually structured the search according to the Population, Intervention, Comparison, and Outcome (PICO) framework:

- Population (P): Patients with CVDs, including arrhythmia, heart failure, ischemic heart disease, cardiomyopathy, and related conditions.
- Intervention (I): Digital twin systems designed for diagnosis, simulation, personalization, monitoring, risk prediction, or therapy planning in cardiology.
- Comparison (C): Not applicable, as the review did not evaluate digital twins against alternative interventions or standard care.
- Outcome (O): Descriptive outcomes related to modeling strategies, data infrastructure, clinical applications, reported clinical impact, implementation barriers, and ethical or governance considerations.

These PICO elements informed the design of our search and eligibility criteria, while the detailed content of the review was organized around 11 thematic RQs (RQ1-RQ11).

All search results were exported to a centralized reference manager and screened using Microsoft Excel. The complete search strings for the databases are provided in Multimedia Appendix 1.

### Eligibility Criteria

Articles were included if they (1) were original empirical research studies, including journal articles, conference papers, and preprints; (2) reported on the development, implementation, or evaluation of digital twin systems in health care; (3) focused on cardiovascular applications, including anatomical, physiological, or functional heart modeling; (4) were related to individualized or personalized medicine, clinical decision-making, or patient-specific therapies; and (5) were published in English and provided a structured abstract.

Articles were excluded if they (1) were review papers, commentaries, editorials, book chapters, or theoretical position pieces; (2) did not focus on cardiovascular systems (eg, neurological or orthopedic digital twins); (3) were not available in full text or lacked an identifiable abstract; (4) were duplicate entries across databases; and (5) were published in languages other than English, including those labeled as “unspecified” or “null.”

These criteria were iteratively refined during the pilot screening of 50 records.

We did not apply the exclusion criteria based on study design, as the aim of this review was to comprehensively synthesize diverse contributions to the digital twin field, including conceptual, technical, and applied studies, without limiting the scope to any particular methodological framework.

### Screening and Article Selection

The initial search returned 330 records. A multistep screening protocol was applied:

- Phase 1 (title and abstract screening): Three reviewers independently screened articles for relevance. Discrepancies were resolved by group discussion and majority vote.
- Phase 2 (eligibility review): Of 44 records identified in phase 1, 2 records were excluded. A final set of 42 articles was included in the synthesis.

Reviewers used a shared Microsoft Excel spreadsheet with predefined drop-down fields for coding decisions. Interreviewer consistency was monitored, and a senior reviewer adjudicated disagreements. The filtering questions used during study selection are presented in Table 1. The complete list of all screened records, along with their inclusion or exclusion status, is provided in Multimedia Appendix 2.

**Table 1.** Filtering questions used during study selection for the systematic review of cardiovascular digital twin research.

Screening question^a^	Decision criteria
Filtering question 1: Does the study relate to digital twins in health care or medicine?	Include if the study discusses digital twins applied in health care contexts.
Filtering question 2: Does the study specifically address the use of digital twins in cardiology?	Include if the study focuses on cardiovascular applications of digital twins.
Filtering question 3: Does the study involve personalized or patient-specific applications in cardiology?	Include if the study discusses patient-specific or precision medicine approaches.

a Each question aligns with predefined inclusion and exclusion criteria applied across titles, abstracts, and full texts.

### RQs and Data Extraction

Data extraction was organized around 11 RQs, which were structured into six thematic categories:

1. Technological foundations: modeling methods (RQ1), mechanistic model types (RQ2), ML algorithms (RQ3), and open-source availability (RQ4)
2. Data infrastructure and visualization: patient-specific data (RQ5) and visualization formats (RQ6)
3. Clinical applications and conditions: clinical use cases (RQ7) and cardiovascular conditions addressed in digital twin studies (RQ8)
4. Clinical impact: reported outcomes and benefits (RQ9)
5. Implementation challenges: technical and validation barriers (RQ10)
6. Ethical considerations: legal, privacy, and governance issues (RQ11)

Each reviewer used a structured extraction form, built in Excel, to code articles across multiple predefined categories (eg, “FEM,” “ECG,” and “Heart Failure”) using a controlled vocabulary. Note fields allowed for contextual elaboration and inductive theme discovery.

Categories were not mutually exclusive, allowing multiple responses per article. The full data extraction form is provided in Multimedia Appendix 3.

### Data Extraction Process

Data extraction followed a structured workflow as follows:

1. Full-text review: Each selected study was fully reviewed to extract methodological details and research contributions.
2. Thematic classification: Studies were assigned to predefined thematic categories based on their focus area and objectives.
3. Double-reviewer validation: Three independent reviewers extracted data; any conflicts were resolved via discussion.
4. Database compilation: Extracted data were compiled into a structured dataset for further analysis.

### Risk of Bias

The risk of bias of the included studies was assessed using the instrument most appropriate for the underlying study design. Three distinct tools were used. First, simulation-based and modeling-oriented studies, such as those involving digital twins, mechanistic models, or computational pipelines, were evaluated using a custom modeling checklist, which was developed to capture methodological risks specific to computational modeling (eg, data representativeness, validation strategy, overfitting, and reproducibility). Second, prediction-modeling studies were appraised using the Prediction Model Risk of Bias Assessment Tool (PROBAST), which evaluates risk of bias across 4 domains: participants, predictors, outcome, and analysis. Finally, observational cohort studies were assessed using the Risk of Bias in Non-Randomized Studies - of Interventions (ROBINS-I), which provides structured domain-level judgments for 7 bias domains, including confounding, selection of participants, classification of interventions, missing data, and outcome measurement.

For all tools, domain-level judgments were assigned according to published guidance or tool-specific documentation. Risk-of-bias assessments were conducted independently by multiple assessors, and any discrepancies were resolved through discussion, with arbitration applied when consensus could not be reached. Domain-level ratings were then synthesized into an overall judgment (low, unclear, or high risk of bias) based on the decision rules recommended for each tool.

Visualization of risk-of-bias judgments was performed using robvis [16], an R package and web application that supports structured display of traffic-light plots and summary plots.

## Results

### Overview

**Figure 1.**
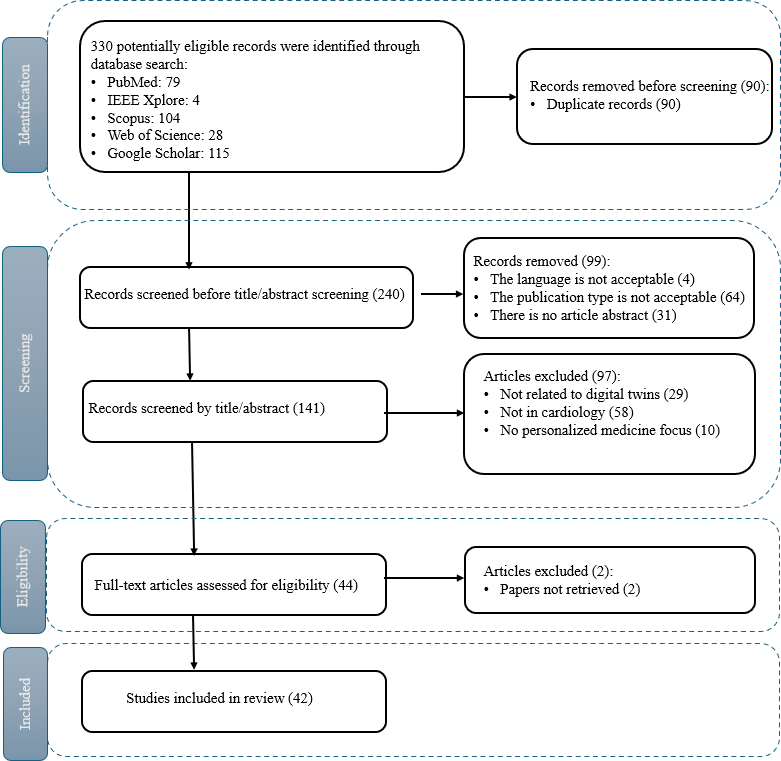
PRISMA (Preferred Reporting Items for Systematic Reviews and Meta-Analyses) 2020 flow diagram illustrating the systematic selection process for cardiovascular digital twin studies. A total of 330 records were retrieved from 5 major databases and screened using predefined eligibility criteria. After removal of duplicates and exclusion of irrelevant or nonoriginal articles, 42 studies were included in the final synthesis for qualitative and quantitative analyses.

We synthesized the findings from 42 original research articles on cardiovascular digital twins. The PRISMA flow diagram of the study selection process is presented in Figure 1. The results were structured around 11 predefined RQs, which were organized into 6 thematic domains: technological foundations, data infrastructure and visualization, clinical applications and conditions, clinical impact, implementation challenges, and ethical considerations. Each subsection follows a format: overview, key insights, and interpretation. For each RQ, we present summary patterns and cite representative studies in the main text. The complete mapping of all studies to the corresponding RQ categories is provided in Multimedia Appendix 4, and the mapping from raw extraction values to the harmonized categories used in the analyses is provided in Multimedia Appendix 5.

Funding sources were reported for a subset of studies and were most often public or academic, with a smaller number supported by mixed public-foundation or public-industry collaborations and relatively few funded solely by industry. Study-level funding details are summarized in Multimedia Appendix 4.

### Technological Foundations (RQ1-RQ4)

We outline the core technical elements of cardiovascular digital twin systems, focusing on modeling strategies (RQ1), types of mechanistic models (RQ2), ML applications (RQ3), and open-source availability (RQ4). Together, these RQs characterized how digital twins were constructed, personalized, and shared, revealing trends in hybrid modeling, the integration of AI, and the challenges in reproducibility.

#### RQ1: What Primary Modeling Approach is Used to Develop Digital Twins?* *

##### Overview

All 42 studies were classified according to their dominant modeling approach: mechanistic, hybrid, or data-driven. These categories reflect the computational core of digital twins, ranging from physics-based simulation to statistical learning and their integration.

##### Key Insights

The key insights are as follows:

- Mechanistic models were the most common (29 studies [8,11,17-43]), and they relied on physics-based formulations (eg, finite element modeling [FEM], electrophysiological simulation, and hemodynamic flow analysis) to generate personalized physiological representations.
- Data-driven models were noted in 7 studies [44-50], and they were primarily based on statistical learning or machine-learning approaches without explicit biophysical constraints.
- Hybrid approaches were the least common (6 studies [51-56]), and they combined mechanistic frameworks with data-driven components, for example, using ML to estimate parameters, extract imaging features, or accelerate computational solvers.

##### Interpretation

The predominance of mechanistic approaches highlights the central importance of physiological interpretability in cardiovascular digital twin development. Studies involving these approaches focus on replicating biophysical behavior with high fidelity, supporting diagnostic and interventional simulation tasks.

Data-driven twins, while less common, demonstrate growing interest in leveraging large clinical datasets for prediction, classification, and risk estimation. Their scope is more limited in scenarios requiring detailed physiological realism.

Hybrid methods illustrate emerging strategies that balance accuracy and computational efficiency. In studies involving these approaches, ML is commonly used to tune physiological parameters, derive boundary conditions from imaging or ECG data, or build surrogate models that reduce the computational cost of mechanistic solvers. In a subset of hybrid digital twin studies [51-56], ML components were typically embedded within a mechanistic framework rather than used in isolation. Across these studies, we observed 3 main integration patterns. First, ML is used for parameter tuning and personalization of mechanistic models, for example, by estimating subject-specific parameters or boundary conditions that are then supplied to a physics-based simulator. Second, ML algorithms are applied for feature extraction from raw clinical data, such as imaging or ECG signals, and the extracted features are subsequently used to initialize or constrain the mechanistic model. Third, in a small number of cases, ML serves as a surrogate or complementary model that approximates the behavior of a more complex mechanistic solver or is combined with mechanistic equations in a joint statistical-mechanistic framework. Together, these hybrid strategies illustrate how data-driven methods can enhance mechanistic digital twins by improving personalization, leveraging high-dimensional data, and reducing computational cost.

### RQ2: If the Model is Mechanistic, What Specific Model Type is Used?

#### Overview

Across the 42 included studies, we identified multiple types of mechanistic models used within cardiovascular digital twin frameworks. Because individual studies often combined more than one formulation, we classified mechanistic components into 9 categories based on their predominant mathematical and physiological characteristics.

#### Key Insights

The key insights are as follows:

- Electrophysiology models were the most common (19 studies [11,17,18,20,21,25,27,28,30,34,35,37,39,41,43,44,46,51,54]). These models typically used monodomain, bidomain, or related reaction-diffusion formulations to simulate cardiac electrical activation, sometimes coupled to downstream mechanical effects.
- FEM-based structural or biomechanical models were used in 10 studies [8,18,19,26,27,29-31,33,37] to represent myocardial or vascular deformation, geometry, and stress-strain behavior.
- Electromechanical models, which explicitly couple electrical activation with tissue mechanics, were identified in 8 studies [22,26,30,32,33,52,53,55]. They supported integrated simulation of excitation-contraction processes.
- Simplified or system-level models, which are most often lumped-parameter formulations, were noted in 7 studies [24,29-31,33,36,53]. They provided compact descriptions of global hemodynamics or chamber-level dynamics, particularly when large-scale or long-duration simulations were required.
- Multiscale models were reported in 7 studies [11,22,30,33,41,44,53], linking processes across spatial or temporal scales (eg, from cellular electrophysiology to organ-level function).
- Computational fluid dynamics (CFD) models were used in 3 studies [40,42,55] to simulate blood flow and pressure distributions in chambers or great vessels. An additional 4 studies [19,23,24,56] used other mechanistic formulations (eg, specialized anatomical or biophysical models), and 1 study [42] used a surrogate mechanistic model that approximated a more complex solver. In 1 study [38], mechanistic modeling was reported, but the specific model type was not clearly described.

#### Interpretation

Taken together, the results show that electrophysiology-focused models form the backbone of mechanistic digital twin development in cardiology, with FEM-based structural models, lumped-parameter and multiscale formulations, and CFD models used in complementary roles. This diversity of model types illustrates how digital twin frameworks combine detailed biophysical fidelity with system-level abstractions to address specific clinical questions and RQs.

### RQ3: If the Model Includes ML or AI, What Specific Algorithms are Applied?

#### Overview

Among the 42 reviewed studies, some explicitly reported using ML or AI techniques within the digital twin framework, while in others, the use or type of ML was absent or not clearly specified. Because several studies combined more than one method, we grouped algorithms into broad families, including DL, Bayesian methods, optimization algorithms, classical (statistical) ML, and other ML approaches.

#### Key Insights

The key insights are as follows:

- DL was the most frequently reported family of methods (9 studies [27,34,44-46,48,49,53,54]). These approaches included architectures, such as convolutional neural networks, neural operators, latent neural ordinary differential equation models, and related deep architectures, used for tasks like feature extraction, representation learning, or surrogate modeling.
- Bayesian methods were used in 5 studies [17,19,25,37,51], typically in the form of approximate Bayesian computation, Bayesian optimization, or Gaussian process–based models for probabilistic parameter estimation and uncertainty quantification.
- Optimization algorithms were noted in 4 studies [19,21,36,51]. These approaches included gradient-based schemes and metaheuristics that were used to tune model parameters, personalize simulations, or search over high-dimensional design spaces; in some cases, these optimizers were tightly integrated with Bayesian frameworks.
- Classical ML methods were identified in 2 studies [50,56]. These approaches included techniques, such as decision tree and logistic or linear regression, to model interpretable relationships between inputs and outcomes.
- Regression was explicitly highlighted as the primary approach in 1 of the studies [50]. One study used other ML strategies that did not fit neatly into the above categories but still relied on data-driven learning to support digital twin construction or personalization [46].

#### Interpretation

Overall, DL has emerged as the dominant explicitly reported ML family in cardiovascular digital twin research, supporting tasks such as feature extraction, surrogate modeling, and high-dimensional inference. Bayesian and optimization-based methods play a complementary role by enabling parameter estimation and uncertainty-aware personalization. Classical ML and regression, although less common, provide more interpretable models in selected use cases.

**Figure 2.**
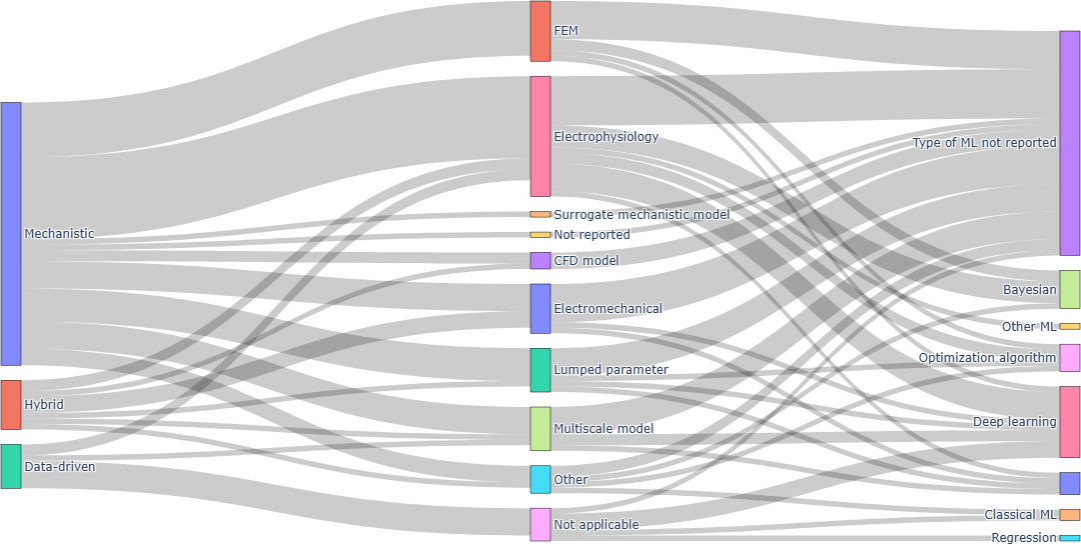
Relationships among modeling approaches, mechanistic model types, and machine learning (ML) or artificial intelligence (AI) methods in cardiovascular digital twin studies. Sankey diagram summarizing links among primary modeling approaches (research question [RQ] 1), mechanistic model types (RQ2), and ML or AI algorithm families (RQ3) across the 42 original research articles on cardiovascular digital twins included in this systematic review. The left column shows the dominant modeling approach for each study (mechanistic, hybrid, or data-driven). The middle column groups mechanistic model types into electrophysiology, finite element modeling (FEM), lumped parameter, electromechanical, computational fluid dynamics (CFD), other mechanistic models, and “not reported.” The right column shows ML or AI families (deep learning, Bayesian methods, optimization algorithms, classical ML, regression, other ML, and “type of ML not reported”). The width of each flow is proportional to the number of studies combining the corresponding categories.

Figure 2 provides an integrated visualization of how primary modeling approaches, mechanistic model types, and ML or AI families co-occur across the included studies.

### RQ4: Is the Framework or Model That is Created or Used Open-Source?

#### Overview

We evaluated the extent to which cardiovascular digital twin frameworks were shared as open-source resources. Code availability is a key indicator of scientific transparency and reproducibility, enabling independent validation and extension by other researchers and clinicians.

#### Key Insights

The key insights are as follows:

- Across the 42 included studies, 16 explicitly reported that their framework or model was available as open-source code [17-20,25,26,30,35,37,38,41,48,51,53,54,56].
- Two studies clearly stated that the code was not publicly released or that the implementation was proprietary [22,39].
- For the remaining 24 studies, code availability was either not mentioned or not described in sufficient detail to determine whether the implementation was accessible. Thus, less than half of the studies (16/42, 38%) provided explicit evidence of open-source sharing, and in many cases, information on code availability was incomplete.

#### Interpretation

Despite increasing attention to reproducibility and open science, most studies in this review did not make their digital twin implementations publicly available. A lack of open-source code hinders transparency, reproducibility, and reusability. The few repositories that were shared provide valuable resources and serve as exemplars for future cardiovascular digital twin research.

### Data Infrastructure and Visualization (RQ5 and RQ6)

The design and utility of cardiovascular digital twin systems depend heavily on how patient-specific data are structured, how outputs are visually communicated, and who the intended users are. This section addresses RQ5 and RQ6 by examining the types of data used to build or personalize digital twins, the formats used to present model outputs, and the target users of these visualizations. Together, these elements shaped the usability, interpretability, and clinical relevance of digital twin systems in practice.

#### RQ5: What Types of Patient-Specific Data are Used to Build or Personalize Digital Twins?

##### Overview

Patient-specific data underpin cardiovascular digital twin systems by enabling individual-level modeling. We explored the distinct categories of data used to personalize these models, ranging from electrical signals and anatomical imaging to omics and wearable-derived data. To facilitate interpretation, the data were grouped into consistent, semantically meaningful categories.

##### Key Insights

The key insights are as follows:

- Imaging data were the most commonly used (32 studies [8,11,17-19,21,22,24-27,29-31,33-40,42-44,46,48,49,51,53-55]). These data typically included modalities, such as MRI, CT, and other structural imaging, used to reconstruct patient-specific anatomy. Echocardiography was explicitly reported in 2 of these studies as a dedicated imaging source.
- Signal-based electrical data, primarily ECG, were used in 18 studies [8,17,21,25,27,33,36-39,43,46,48-51,53,54], reflecting its central role in modeling cardiac electrophysiology and conduction abnormalities.
- Vital signs were used in 12 studies [17,24,26,28,29,31,33,45,48,49,53,55], and demographics, such as age and sex, were reported in 9 studies [17,24,27,28,33,45-48], often to support model initialization, risk stratification, or cohort characterization.
- More detailed clinical information appeared in several categories: omics data were used in 4 studies [22,23,49,55], lab results were used in 4 studies [22,28,49,55], and general clinical data (such as clinical histories and visit summaries) were used in 3 studies [49,52,55]. Diagnosis [33,47,48] and treatment-related data [33,47,48] (eg, information on interventions or therapies) were each reported in 3 studies.
- Sensor-based and longitudinal monitoring information was less common: 3 studies used data from sensors [46,49,55], and 2 studies used activity tracker data [45,49], illustrating the early integration of wearable or home-based measurements into digital twin personalization. Synthetic patient data were explicitly used in 1 study [56].

##### Interpretation

Overall, there is a strong reliance on imaging and ECG data to define anatomy and electrophysiological behavior in cardiovascular digital twins, complemented by vital signs and demographic information for basic personalization. Omics, lab results, richer clinical records, and wearable or sensor-derived data are beginning to appear but remain less common, suggesting that truly multimodal, longitudinal personalization is still emerging. The presence of synthetic and other less conventional data sources indicates ongoing experimentation with alternative data strategies.

### RQ6: What is the Primary Format Used to Visually Present Digital Twin Outputs?

#### Overview

We examined how digital twin outputs were visualized in cardiovascular studies, an essential aspect for interpretation, user interaction, and eventual clinical integration. Each study could use more than one visualization format, so outputs were classified into standard categories such as static figures, anatomical renderings, tables, dashboards, and interactive media.

#### Key Insights

The key insights are as follows:

- Static figures were the most common visualization format (41 studies [8,11,17-47,49-56]). These typically included plots, error curves, comparative graphics, and screenshots of simulations, and were primarily designed for inclusion in scientific publications.
- Two- or three-dimensional anatomical views were reported in 27 studies [8,11,17-19,21,22,24-27,29-31,33-35,37,39,40,42-44,46,51,54,55], where patient-specific geometries or simulated fields (eg, activation times, strain, and flow patterns) were mapped onto cardiac or vascular structures. These views served to visually link model predictions to underlying anatomy.
- Tabular formats were used in 7 studies [19,22,24,35,36,45,49] to report numerical outputs such as performance metrics, parameter values, and summary statistics.

#### Interpretation

Overall, visualization of cardiovascular digital twins remains dominated by static, publication-oriented formats such as figures and anatomical snapshots, with limited support for dynamic, interactive, or dashboard-based exploration. While anatomical views help contextualize outputs in patient-specific geometry, the scarcity of dashboards, animations, and interactive interfaces suggests that user-centric and real-time visualization capabilities are still underdeveloped. Enhancing interactive and clinically oriented visualization tools may be crucial for translating digital twins from research prototypes into practical decision-support systems.

### Clinical Applications (RQ7 and RQ8)

We explored how digital twins were applied in clinical cardiology (RQ7) and which cardiovascular conditions they targeted (RQ8). It highlighted current use cases, such as diagnosis, planning, and monitoring, and categorized the conditions based on thematic grouping identified during full-text analysis.

#### RQ7: What is the Main Clinical Application or Use Case of Digital Twin Systems?

##### Overview

We explored the primary clinical applications of cardiovascular digital twin systems, revealing the core motivations behind their development and deployment. Use cases ranged from therapy planning and risk prediction to monitoring, drug testing, and more exploratory clinical applications. Individual studies could contribute to multiple application categories.

##### Key Insights

The key insights are as follows:

- Therapy planning was the most common application (28 studies [8,11,17,19,20,22-25,29-34,36-39,41,45-47,49,51,54-56]). In these studies, digital twins were used to support the selection, personalization, or optimization of interventions, including device configuration, ablation strategies, or other patient-specific treatment plans.
- Risk prediction was noted in 11 studies [20,28,40,41,46-50,52,55], where digital twins were used to estimate the likelihood of adverse events, treatment responses, or disease trajectories, often to support patient stratification. Diagnosis-focused applications were identified in 7 studies [11,45,46,48-50,54], using digital twins to assist in identifying underlying pathophysiology or classifying clinical conditions.
- Surgical and device simulation was reported in 6 studies [36,38,42,46,51,55], in which digital twins provided virtual testbeds to explore procedural strategies or evaluate device performance in patient-specific anatomies. Another 6 studies used digital twins for drug testing [17,20,28,32,36,37].
- Monitoring applications were noted in 6 studies [45,48-50,52,55], where digital twins contributed to disease tracking or follow-up by integrating longitudinal data or repeated assessments. Disease progression modeling was explicitly highlighted in 3 studies [36,42,55], and a single study focused primarily on prognosis [55].

##### Interpretation

Overall, cardiovascular digital twins are most frequently positioned as tools for therapy planning and risk prediction, emphasizing their role in personalizing and optimizing clinical interventions. Diagnosis, surgical or device simulation, drug testing, and monitoring collectively demonstrate a broad range of applications along the care pathway, from early risk assessment to procedural planning and follow-up. As digital twin technologies mature, a clearer definition and reporting of clinical applications will be important for understanding their real-world impact.

### RQ8: What Cardiovascular Conditions Are Studied Using Digital Twin Systems?

#### Overview

We examined the range of cardiovascular conditions addressed by digital twin systems, providing a disease-centered perspective on where digital twin technologies are currently being applied. Conditions were grouped into clinically meaningful categories, and the classification was reviewed by a physician on the research team to ensure clinical relevance and consistency.

#### Key Insights

The key insights are as follows:

- Arrhythmia was the most frequently studied condition (13 studies [8,11,18,20,25,28,30,34,38-41,43]). The studies predominantly focused on atrial fibrillation and other rhythm disorders, reflecting the suitability of digital twins for simulating electrophysiological mechanisms and guiding rhythm-related interventions.
- Heart failure was investigated in 9 studies [33,36,38,41,48,51-53,55], often in the context of global cardiac function, ventricular remodeling, or device-based therapies. Cardiomyopathies, including hypertrophic cardiomyopathy and other structural myocardial diseases, were the primary focus in 5 studies [19,22,32,35,44], where digital twins were used to explore patient-specific mechanics and electrophysiology.
- Six studies centered on healthy or control populations [17,43,46,49,54,56], using digital twins to represent normal physiology, establish reference behaviors, or provide baselines for comparison with diseased states. Aortic disease was the focus in 3 studies [26,29,42], typically involving patient-specific modeling of the aorta for flow, wall stress, or device evaluation.

#### Interpretation

The distribution shows a strong emphasis on arrhythmia and heart failure, conditions in which digital twins can leverage detailed electrophysiological and hemodynamic modeling to support diagnosis, therapy planning, and risk assessment. Cardiomyopathies, aortic disease, and valvular disease are also emerging areas of application, particularly where structural and flow abnormalities can be represented in patient-specific models. By contrast, hypertension, atherosclerosis, and some other common cardiovascular conditions are only sporadically represented, and several studies do not clearly specify the underlying disease focus. These gaps suggest opportunities for expanding digital twin applications into a broader spectrum of cardiovascular conditions and for improving the clarity of disease reporting in future work.

**Figure 3.**
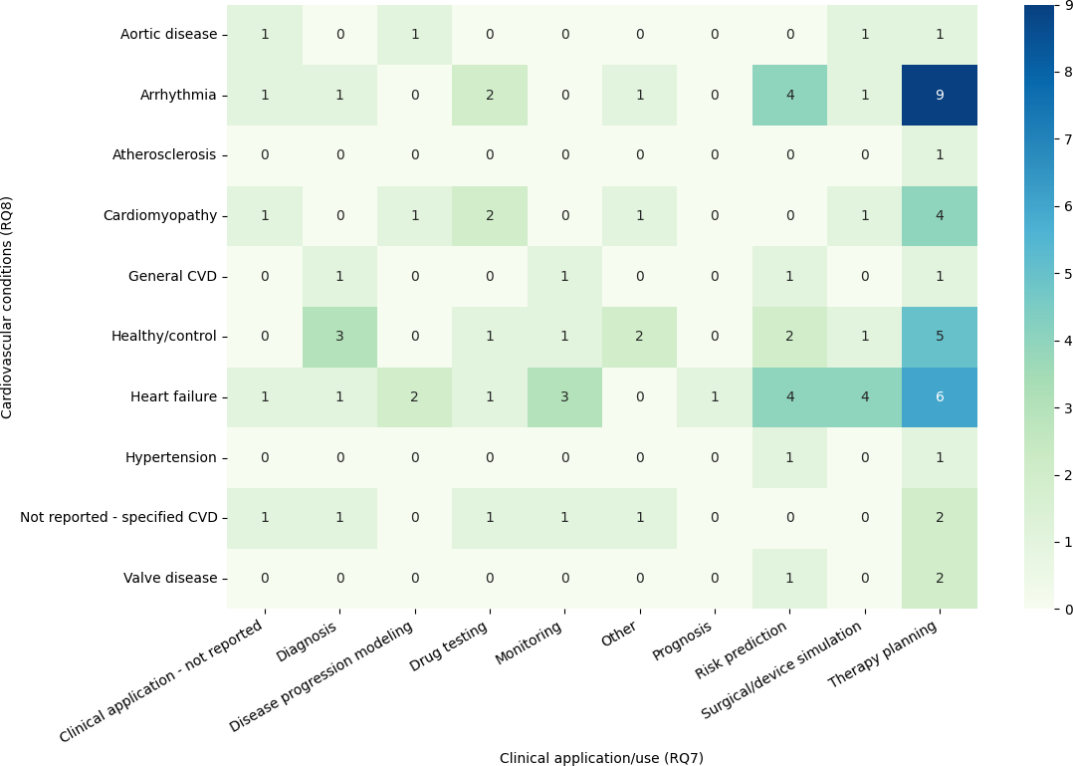
Heatmap of cardiovascular conditions (research question [RQ] 8) versus clinical applications (RQ7) in cardiovascular digital twin studies. Rows show the primary cardiovascular condition modeled (eg, arrhythmia, heart failure, cardiomyopathy, aortic and valve disease, hypertension, atherosclerosis, general cardiovascular disease [CVD], healthy/control, and not reported). Columns show the main clinical applications (eg, diagnosis, disease progression modeling, drug testing, monitoring, prognosis, risk prediction, surgical or device simulation, and therapy planning). Cell color and numbers indicate how many of the 42 included studies reported each condition-application combination (darker cells indicate a higher number of studies).

Figure 3 summarizes how clinical applications are distributed across cardiovascular conditions. As shown in Figure 3, therapy planning and risk prediction are concentrated in arrhythmia and heart failure, whereas other conditions and applications are represented by only a small number of studies, underscoring the uneven distribution of digital twin work across CVDs.

### Impact on Clinical Practice (RQ9)

We identified the reported clinical benefits of cardiovascular digital twin systems, including improved accuracy, personalization, therapy planning, and patient outcomes. The findings were organized into key impact categories to highlight where digital twins showed practical value in care delivery.

#### RQ9: What Clinical Impacts are Reported as a Result of Using Digital Twins?

##### Overview

We examined the concrete clinical or clinically relevant impacts attributed to cardiovascular digital twin systems. Rather than focusing on intended use alone, we captured reported effects where the use of a digital twin was described as influencing decision-making, therapy, diagnostic performance, or other aspects of care. Reported impacts were grouped into categories such as improved decision-making, therapy-related benefits, increased accuracy, and other specific outcomes.

##### Key Insights

The key insights are as follows:

- Improved decision-making was the most frequently reported impact (19 studies [22,24,28,30-34,36,38-41,47-49,51,55,56]). In these cases, digital twins were described as helping clinicians compare alternative strategies, understand patient-specific mechanisms, or select interventions with greater confidence.
- Therapy-related impacts were reported in 18 studies [19,22-25,32,34,36,38-41,45,47-49,51,55], including optimization of device settings, refinement of ablation targets, adjustment of pharmacologic regimens, and more tailored procedural planning based on virtual simulations.
- Increased accuracy was explicitly identified in 6 studies [31,45,48,49,54,55], referring to improvements in predictive performance, better correspondence between simulations and measured clinical data, or more faithful reproduction of patient-specific physiology. Two studies [49,50] reported a faster diagnostic process, where digital twin–supported workflows were associated with quicker identification or clarification of clinical conditions.

**Figure 4.**
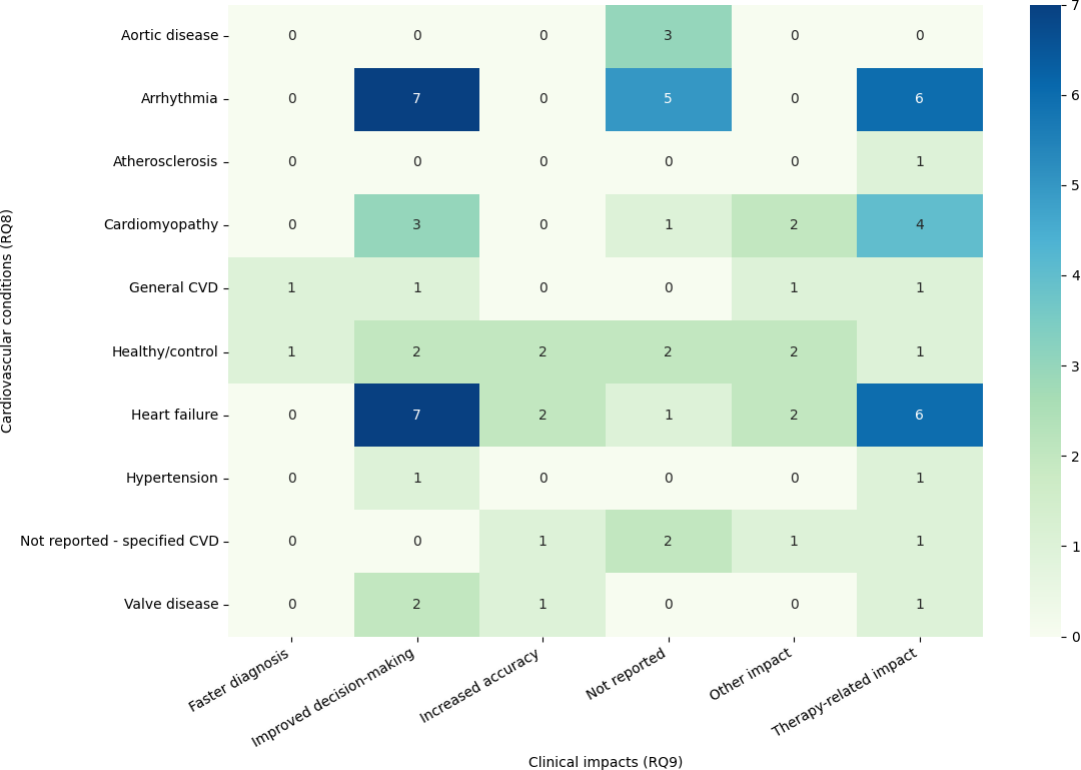
Heatmap of cardiovascular conditions (research question [RQ] 8) versus reported clinical impacts (RQ9) in cardiovascular digital twin studies. Rows represent the primary cardiovascular condition modeled by the digital twin (eg, arrhythmia, heart failure, cardiomyopathy, aortic and valve disease, hypertension, atherosclerosis, general cardiovascular disease [CVD], healthy/control, and not reported). Columns represent impact categories reported by study authors (faster diagnosis, improved decision-making, increased accuracy, impact not reported, other impact, and therapy-related impact). Cell color and numbers indicate how many of the 42 included studies reported each condition-impact combination (darker cells indicate a higher number of studies).

Figure 4 illustrates how reported clinical impacts are distributed across cardiovascular conditions. Improved decision-making and therapy-related impacts were concentrated in arrhythmia and heart failure, whereas many other condition-impact combinations were represented by only one or two studies, highlighting the uneven evidence base across disease areas.

##### Interpretation

The most commonly reported benefits of cardiovascular digital twins relate to improved clinical decision-making and therapy-related impacts, suggesting that these systems are beginning to influence how clinicians choose and personalize interventions. Explicit gains in accuracy and diagnostic speed are less frequently reported but point toward the quantitative advantages of model-based approaches when they are carefully evaluated. At the same time, the substantial number of studies with no clearly articulated clinical impact indicates that much of the current literature remains focused on technical feasibility and validation rather than demonstrated downstream effects on care processes or patient outcomes. Strengthening the evidence base around measurable clinical benefits, such as improved decision quality, optimized therapy, and better outcomes, will be essential for wider clinical adoption.

**Figure 5.**
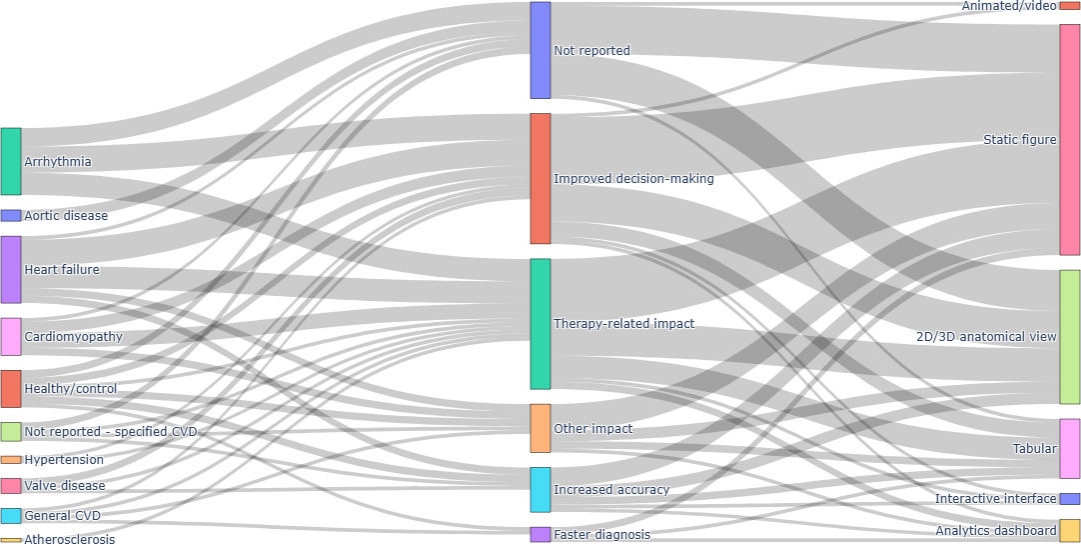
Relationships among cardiovascular conditions, reported clinical impacts, and visualization formats in cardiovascular digital twin studies. Sankey diagram summarizing links among cardiovascular conditions (research question [RQ] 8), reported clinical impacts (RQ9), and primary visualization formats (RQ6) across the 42 original research articles on cardiovascular digital twins included in this systematic review. The left column shows the main conditions modeled by the digital twins (eg, heart failure, arrhythmia, valve disease, cardiomyopathy, hypertension, atherosclerosis, general cardiovascular disease [CVD], and healthy/control populations). The middle column displays impact categories reported by the authors (eg, improved decision-making, therapy-related impact, increased accuracy, faster diagnosis, and other impact). The right column shows the dominant visualization formats used to present model outputs (static figures, 2D/3D anatomical views, tabular displays, analytics dashboards, animated/video outputs, and interactive interfaces). The width of each flow is proportional to the number of studies combining the corresponding categories.

Figure 5 shows that improved decision-making is the dominant reported impact across most cardiovascular conditions, particularly heart failure and arrhythmia, and that these impacts are almost always communicated through static figures and 2D or 3D anatomical views rather than dashboards, animations, or interactive interfaces. Therapy-related impacts and gains in accuracy are more sparsely reported and similarly rely on conventional publication-style visualizations, underscoring the limited development of user-facing, real-time visual tools, even in high-risk clinical scenarios.

### Barriers to Implementation and Ethical Considerations (RQ10 and RQ11)

We examined the key challenges limiting the adoption of cardiovascular digital twins, including technical barriers (RQ10) and ethical or legal concerns (RQ11). These issues highlighted the need for improved scalability, transparency, and responsible use in clinical settings.

#### RQ10: What Limitations or Practical and Technical Barriers are Described?

##### Overview

We identified the limitations and implementation barriers of cardiovascular digital twin systems as reported by the included studies. Rather than listing every individual issue, reported limitations were grouped into conceptually meaningful categories, such as model assumptions, computational constraints, data-related challenges, and integration or usability problems. This categorization helped highlight systemic obstacles that recur across the field.

##### Key Insights

The key insights are as follows:

- Model assumptions and structural simplifications were the most frequently cited limitations (26 studies [8,11,17,19-22,24,25,27-33,35,36,39,41,43,44,46,47,51,53]). These concerns included oversimplified anatomy or physiology, restrictive boundary conditions, and reduced model complexity that may limit generalizability or omit important mechanisms.
- Computational cost was highlighted in 21 studies [11,17,21,22,26,27,29-31,33,36,37,39,41,42,46,49,51,53,54,56], where authors noted long simulation times, high hardware requirements, or overall computational burden that can impede large-scale studies and real-time or near–real-time clinical use.
- Data-related challenges were prominent, with 16 studies [19,22,25,27-30,36,39,40,43,45,47,50,55,56] reporting issues with data quality or availability, such as incomplete or noisy clinical inputs, limited access to high-resolution or longitudinal data, and difficulties in acquiring truly personalized datasets. Limited validation was also mentioned in 16 studies [24,25,27-29,31,33-36,39,40,45,47,53,55], reflecting concerns about small sample sizes, restricted cohorts, synthetic data, or a lack of robust testing in real-world clinical environments.
- More specific barriers included a lack of real-time performance in 5 studies [33,42,50,54,55], indicating that even when models were accurate, their latency or compute demands were not compatible with time-sensitive clinical workflows. Workflow integration problems were identified in 4 studies [30,47,51,55], focusing on the challenges of embedding digital twins into existing clinical systems and processes. Clinician usability challenges were noted in 3 studies [45,49,55], where interfaces or outputs were considered difficult to interpret or not well aligned with clinical practice. High infrastructure cost was noted in 2 studies [49,53], and data security or privacy concerns were explicitly mentioned in 1 study [49].

##### Interpretation

The most common limitations—strong model assumptions, high computational cost, and data and validation constraints—reflect the technical and methodological complexity of deploying cardiovascular digital twins in practice. Simplifying assumptions and limited data can undermine generalizability, while computational burden and lack of real-time performance can restrict clinical usability. Integration issues, usability challenges, infrastructure demands, and security concerns, though mentioned less often, highlight important practical barriers that will become more pressing as digital twins move closer to clinical deployment. Addressing these limitations through improved model design, better data infrastructure, efficient algorithms, and user-centered integration will be essential for scalable, clinically viable digital twin systems.

### RQ11: What Legal, Ethical, or Data Governance Issues are Raised Regarding Digital Twins?

#### Overview

We explored the ethical, legal, and data governance concerns raised in studies involving cardiovascular digital twin systems. Potential issues included privacy protection, regulatory compliance, informed consent, algorithmic transparency, and fairness. Reported concerns were grouped into categories to highlight common themes and gaps in current practice.

#### Key Insights

The key insights are as follows:

- Only a small subset of studies explicitly discussed legal, ethical, or governance issues. Privacy and data protection were the most frequently mentioned topics, identified in 4 studies [47,49,50,55], with references to compliance frameworks, such as General Data Protection Regulation (GDPR) and Health Insurance Portability and Accountability Act (HIPAA), and concerns about safeguarding sensitive patient data in the context of high-dimensional digital representations.
- Some studies raised other specific issues. Two studies discussed ethical or legal challenges in general terms [30,47], while another identified potential algorithmic bias, described problems or open questions around informed consent, and highlighted concerns about model transparency and the need for explainable or interpretable digital twin behavior [55].

#### Interpretation

Overall, explicit discussion of legal, ethical, and data governance aspects remains limited in the cardiovascular digital twin literature. While privacy and regulatory compliance are beginning to appear as concrete concerns, far fewer studies engage with broader questions around algorithmic bias, transparency, informed consent in the context of complex modeling, or downstream legal responsibilities. As digital twin systems move closer to clinical deployment and real-world decision support, more systematic attention to these dimensions, including fairness, accountability, liability, and data stewardship, will be critical to ensure trustworthy and responsible adoption.

### Risk of Bias Assessment

The risk of bias was assessed for all included studies using the tool corresponding to the underlying study design. Among the 42 studies evaluated, 38 were computational or simulation-based studies assessed using the custom modeling checklist [8,11,17-38,40-46,48,51-56], 2 were prediction-modeling studies evaluated using the PROBAST [49,50], and 2 were observational cohort studies evaluated using the ROBINS-I [39,47].

Table 2 summarizes the distribution of overall risk-of-bias judgments across the 3 tools. For simulation and digital twin modeling studies, “unclear” was the most frequent overall rating (22/38, 58%), followed by “high risk” (16/38, 42%). The domains contributing most frequently to elevated risk included data representativeness, validation strategy, and sample size/overfitting. No modeling study received an overall low-risk judgment, reflecting commonly observed methodological limitations in data availability, external validation, and reproducibility practices across computational literature.

**Table 2.** Summary of overall risk-of-bias judgments across the included studies.

Tool	Total studies (N=42), n	Unclear risk, n (%)	High risk, n (%)
Custom modeling checklist	38	22 (58)	16 (42)
PROBAST^a^	2	0 (0)	2 (100)
ROBINS-I^b^	2	1 (50)	1 (50)

a PROBAST: Prediction Model Risk of Bias Assessment Tool.

b ROBINS-I: Risk of Bias in Non-Randomized Studies - of Interventions.

Both prediction-modeling studies assessed with the PROBAST were rated as having a high risk of bias, predominantly due to concerns in the analysis and outcome domains, including insufficient handling of model calibration, unclear predictor specification, and absence of prespecified analysis protocols.

Among the 2 observational cohort studies evaluated using the ROBINS-I, one was judged as having a high risk of bias, primarily due to serious confounding and selective reporting, while the other was rated as unclear.

**Figure 6.**
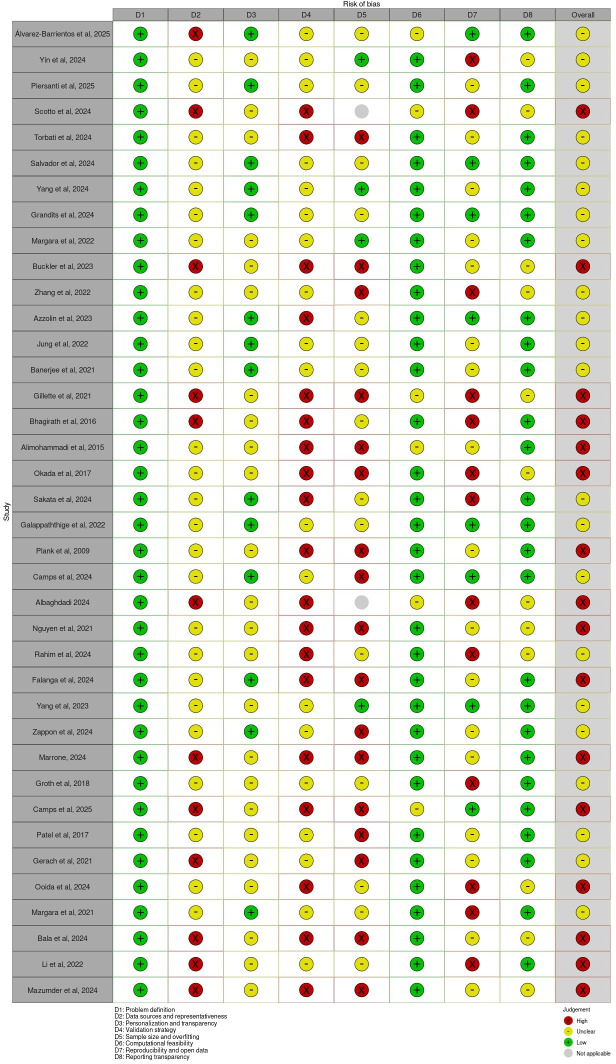
Risk of bias assessment (traffic-light plot) for modeling studies [8,11,17-38,40-46,48,51-56]. Traffic-light plot for the 38 simulation/digital twin modeling studies assessed using the custom modeling checklist. Domain-level judgments are categorized as low, unclear, or high. The plot has been generated using the robvis tool [16].

Figures6  7 present the traffic-light and summary plots, respectively, for all 38 modeling studies assessed using the custom modeling checklist. These visualizations highlight consistent methodological limitations across key domains, particularly external validation and representativeness of data inputs. Traffic-light and summary plots for the PROBAST and ROBINS-I assessments are provided in Figures8  9, respectively.

A structured visualization workflow was implemented using the robvis tool, which standardizes the graphical representation of domain-level and overall judgments and supports transparent reporting of risk-of-bias evaluations.

**Figure 7.**
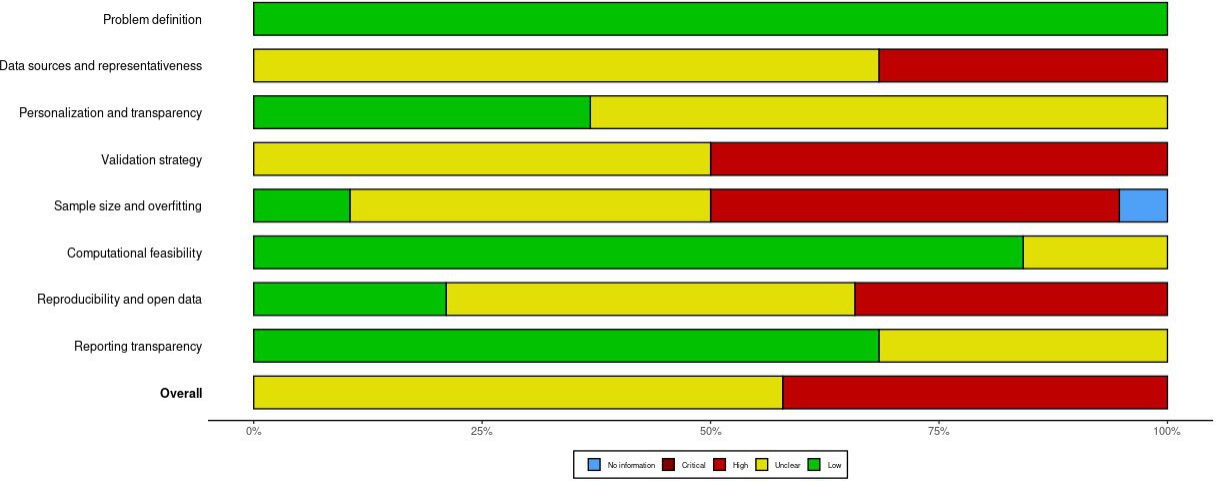
Risk of bias assessment (summary plot) for modeling studies [8,11,17-38,40-46,48,51-56]. Summary plot for the 38 simulation/digital twin modeling studies assessed using the custom modeling checklist. Domain-level judgments are categorized as low, unclear, or high. The plot has been generated using the robvis tool [16].

**Figure 8.**
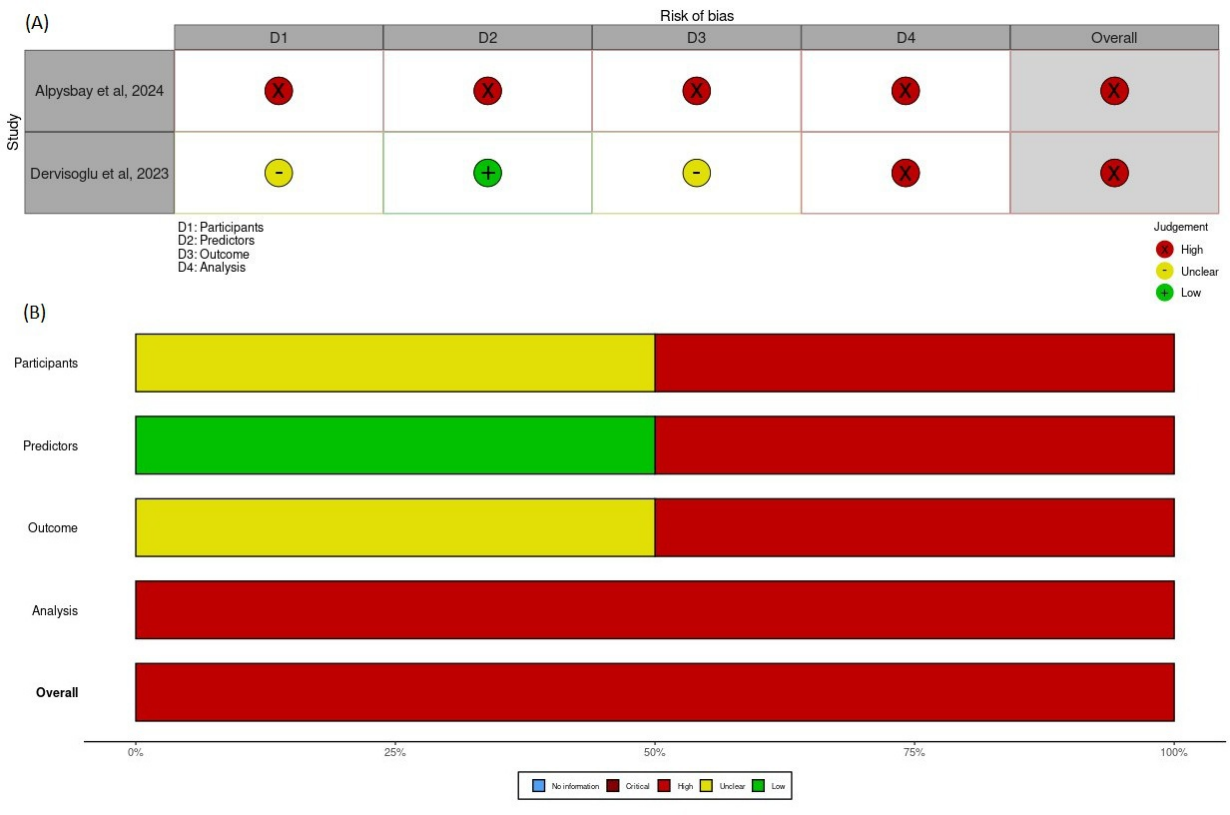
Risk of bias assessment for prediction-modeling studies (Prediction Model Risk of Bias Assessment Tool [PROBAST]) [49,50]. Traffic-light plot (A) and summary plot (B) for the 2 prediction-modeling studies evaluated using the PROBAST instrument. Judgments are shown across the 4 PROBAST domains (participants, predictors, outcome, and analysis) and the overall study-level rating. Visualizations are created using the robvis tool [16].

**Figure 9.**
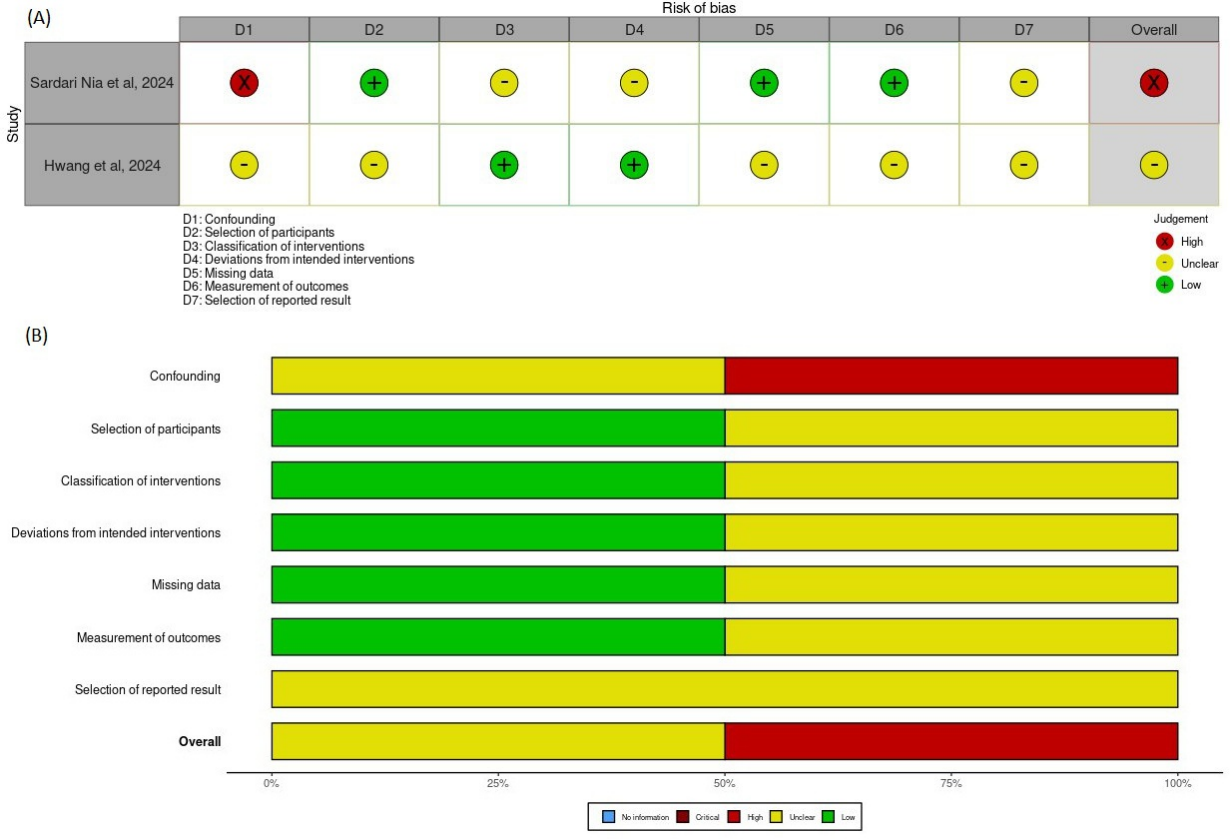
Risk of bias assessment for observational cohort studies (Risk of Bias in Non-Randomized Studies - of Interventions [ROBINS-I]) [39,47]. Traffic-light plot (A) and summary plot (B) for the 2 observational cohort studies evaluated using the ROBINS-I tool. Judgments are shown across the 7 ROBINS-I bias domains and the overall risk-of-bias rating. Visualizations are created using the robvis tool [16].

## Discussion

### Principal Findings

This systematic review synthesized findings from 42 studies and showed that cardiovascular digital twin technology is progressing rapidly but remains largely preclinical and methodologically heterogeneous. Most systems relied on mechanistic models, with a smaller subset incorporating explicit ML or hybrid mechanistic–data-driven designs. Applications clustered around arrhythmia (13/42, 31%), heart failure (9/42, 21%), and therapy planning (28/42, 67%), yet relatively few studies reported real-world clinical deployment, rigorous validation (16/42, 38%), or patient-level outcomes, underscoring the gap between technical innovation and routine clinical use.

Across 11 RQs spanning modeling foundations, data infrastructure, clinical applications, clinical impact, and implementation challenges, the review identified steady technical progress alongside persistent limitations in data quality, external validation, usability, and ethical governance. Our structured risk-of-bias assessment further highlighted that most modeling and prediction studies were judged as having unclear or high risk of bias, particularly in relation to data representativeness, validation strategies, and analysis procedures. Together, these findings suggest that cardiovascular digital twins are scientifically promising but not yet ready for widespread clinical translation.

### Technological Foundations and Modeling Strategies

Mechanistic models form the backbone of current cardiovascular digital twins. Electrophysiology, finite-element structural modeling, lumped-parameter formulations, multiscale frameworks, and CFD-based flow simulations were frequently combined to capture different physiological scales and processes. The predominance of mechanistic approaches reflects the central importance of physiological interpretability and explicit biophysical assumptions in cardiology, where understanding causal mechanisms is often as important as prediction performance.

Hybrid designs and explicit ML or AI integrations were present but less common than might be expected given the broader trends in digital health. Only a minority of studies (18/42, 43%) clearly described ML algorithms, with DL (9/42, 21%), Bayesian methods (5/42, 12%), and optimization algorithms (4/42, 10%) used for tasks such as parameter estimation, feature extraction, surrogate modeling, and uncertainty quantification. Many other papers referred to “ML” or “AI” without specifying algorithm families or training procedures, limiting reproducibility and comparability. Open-source dissemination was also limited; less than half of the studies (16/42, 38%) provided accessible code, constraining independent verification, reuse, and benchmarking.

### Data Infrastructure and Visualization

Personalization of cardiovascular digital twins relied heavily on structural imaging (32/42, 76%) and electrical signals (18/42, 43%). Imaging data (most often MRI or CT, with occasional echocardiography) enabled patient-specific anatomical reconstruction, while ECG and related electrical measurements supported modeling of activation patterns and conduction abnormalities. Vital signs (12/42, 29%) and demographic variables (9/42, 21%) were commonly used as basic covariates, but richer data sources appeared in only a subset of studies (omics: 4/42, 10%; lab results: 4/42, 10%; detailed clinical records: 3/42, 7%; and wearable/sensor streams: 4/42, 10%). This pattern suggests that many digital twins remain anchored in traditional imaging and electrophysiology pipelines, with multimodal, longitudinal data integration still in an early stage.

Visualization practices were predominantly static and publication-oriented. Most studies communicated digital twin outputs through static figures (41/42, 98%), anatomical overlays (27/42, 64%), or tables summarizing simulation results (7/42, 17%). Only a few described dashboards, dynamic animations, or interactive interfaces that would support real-time exploration or clinical decision-making. As a result, the “front end” of many digital twin systems remains geared toward researchers rather than clinicians or patients, which may hinder adoption even when the underlying models are sophisticated.

### Clinical Applications and Target Conditions

Clinically, cardiovascular digital twins were most frequently positioned as tools for therapy planning (28/42, 67%), risk prediction (11/42, 26%), and monitoring (6/42, 14%), with additional roles in diagnosis (7/42, 17%), surgical or device simulation (6/42, 14%), and drug testing (6/42, 14%). Conditions, such as atrial fibrillation and other arrhythmias (13/42, 31%), heart failure (9/42, 21%), cardiomyopathy (5/42, 12%), and aortic disease (3/42, 7%), were most commonly represented, reflecting both their high burden and the suitability of these conditions for simulation-based assessment. Several studies (6/42, 14%) used digital twins to model healthy or control populations, providing physiological baselines and enabling comparison with diseased states.

At the same time, important cardiovascular domains remain underrepresented. Hypertension, atherosclerosis, congenital heart disease, and some valvular pathologies appeared relatively rarely or were only indirectly addressed, despite their major contribution to global cardiovascular morbidity. Furthermore, in some studies (4/42, 10%), the underlying clinical condition was not clearly specified, blurring the line between generic modeling exercises and disease-focused digital twin applications. This uneven coverage limits our ability to generalize digital twin findings across the broader spectrum of CVD.

### Impact on Clinical Practice

Reported clinical impacts aligned with the conceptual promise of digital twins but were often indirect or inferred. The most commonly cited benefits were improved decision-making (19/42, 45%) and therapy-related impacts (18/42, 43%), including better selection of interventions, refined device configurations, and more personalized procedural planning. Some studies (6/42, 14%) reported increased accuracy of predictions or simulations, and a small number of studies (2/42, 5%) documented faster diagnosis or workflow advantages.

However, very few studies (4/42, 10%) linked digital twin use to robust patient-level outcomes such as mortality, hospitalization, and long-term symptom burden. Most evidence came from retrospective analyses, in silico comparisons, or small proof-of-concept applications rather than prospective, real-world evaluations. Consequently, while digital twins appear to enhance mechanistic understanding and may plausibly improve decision quality, the causal pathway from digital twin use to improved patient outcomes remains largely hypothetical. This observation was reinforced by the risk-of-bias assessments, which highlighted frequent limitations in sample size, external validation, and outcome measurement.

### Barriers to Implementation and Ethical Considerations

Several recurring barriers emerged across the included studies. Strong model assumptions and structural simplifications, while often necessary for tractability, raise questions about generalizability to broader populations or clinical settings. High computational cost and limited real-time performance constrain scalability and integration into time-sensitive workflows, particularly in acute care or interventional environments. Data-quality issues, including incomplete or noisy inputs and limited access to comprehensive, longitudinal datasets, further restrict personalization and increase uncertainty.

Workflow integration and clinician usability remain significant challenges. Only a minority of studies (4/42, 10%) described how digital twin systems might be embedded within electronic health records, imaging systems, or existing decision-support tools, and even fewer studies (3/42, 7%) reported formal usability testing with clinicians. Ethical, legal, and governance issues were discussed explicitly in only a small subset of articles (4/42, 10%), primarily in relation to privacy and data protection frameworks such as GDPR and HIPAA. Isolated studies mentioned algorithmic bias, informed consent, or transparency concerns (1/42, 2%), but systematic engagement with liability, accountability, data ownership, and equity was rare, despite their importance for future clinical deployment.

### Sources and Implications of Heterogeneity

Across the included studies, we observed substantial heterogeneity in how cardiovascular digital twins were conceptualized, implemented, and evaluated. This variability spanned multiple dimensions, including the definition and scope of the “digital twin,” the underlying modeling strategies (eg, electrophysiology, finite-element, lumped-parameter, multiscale, CFD, and hybrid ML-mechanistic designs), the types and combinations of data modalities used for personalization, the clinical applications and disease targets, and the choice of validation approaches and outcome metrics. As a result, the findings are difficult to compare directly across studies, and a quantitative synthesis or meta-analysis is not appropriate. This heterogeneity also limits the generalizability of individual results and makes it challenging to derive standardized performance expectations for cardiovascular digital twins. Future work will benefit from clearer definitions, minimum reporting standards, and shared benchmarks to enable a more systematic comparison and aggregation of evidence.

### Implications and Future Directions

The findings of this review suggest that cardiovascular digital twins are technically promising but not yet consistently validated, standardized, or integrated into routine care. Heterogeneity in modeling approaches, data inputs, validation strategies, and reporting practices limits comparability and makes it difficult to draw firm conclusions about real-world effectiveness.

Future work should focus on strengthening clinical validation in real-world settings, ideally through prospective and multisite studies that link digital twin use to patient outcomes and workflow changes. In parallel, clearer definitions of what constitutes a digital twin; shared performance metrics; and minimum reporting standards for models, data, and validation would support meaningful comparisons and regulatory assessments. Methodological transparency and user-centered design are also essential. Explainable or interpretable modeling pipelines and clinician-oriented interfaces are likely to be critical for trust and adoption.

Finally, ethical and equity considerations need to be addressed proactively. Most existing studies draw on narrow populations and rarely examine algorithmic bias, informed consent for complex modeling, or long-term data governance. Future research should deliberately include diverse populations and care settings; evaluate generalizability across subgroups; and embed privacy protection, transparency, and fair data use into the design and deployment of digital twin systems. Closer collaboration with regulators and health care organizations will be important to ensure that these technical and ethical advances translate into safe, accountable, and clinically useful tools.

### Limitations of This Review

While comprehensive, this review may have missed relevant studies, especially studies published in non-English sources or proprietary implementations outside academic literature. Reporting heterogeneity also limited the comparability of validation and outcome data. As the field evolves rapidly, some emerging developments may not have been captured in the included studies.

Although we conducted a structured risk-of-bias appraisal using a custom modeling checklist for simulation studies, the PROBAST for prediction models, and the ROBINS-I for observational cohort studies, these tools were not originally designed for all types of cardiovascular digital twin research and required judgment-based adaptation. In addition, the substantial heterogeneity in study designs, data sources, and evaluation strategies precludes quantitative synthesis and indicates that our risk-of-bias judgments should be interpreted as broad indicators of methodological robustness rather than definitive ratings for individual studies.

This review was based on searches of major bibliographic databases and Google Scholar but did not include dedicated, systematic searches of clinical trial registries (eg, ClinicalTrials.gov), conference proceedings, or specialized grey-literature repositories (eg, dissertation or technical report databases). Although Google Scholar can index some gray literature and conference outputs, our screening was not designed to comprehensively capture these sources. As a result, ongoing trials, conference-only presentations, and nontraditionally published or proprietary digital twin implementations may be underrepresented in this synthesis.

Finally, the review protocol was not registered on a public platform, such as the Open Science Framework (OSF), which may limit reproducibility and transparency. Future work would benefit from prospective protocol registration to reduce the risk of selective reporting and enhance methodological rigor.

### Conclusion

This systematic review mapped the technological, clinical, and implementation landscape of cardiovascular digital twin systems across 42 original studies. We found that most digital twins are grounded in mechanistic modeling, with limited but growing use of hybrid and AI-driven approaches. Personalization relies predominantly on imaging and electrical signals, and applications are concentrated in therapy planning, risk prediction, and monitoring for arrhythmia and heart failure. Although the reported impacts on decision-making and therapy optimization are promising, evidence for downstream patient-level benefits remains sparse.

At the same time, substantial heterogeneity in model architectures, data modalities, clinical use cases, and validation strategies—combined with incomplete reporting of algorithms, data, and code—limits comparability across studies and precludes quantitative synthesis. Key barriers to clinical translation include strong modeling assumptions; high computational cost; constrained data quality and availability; and limited real-time performance, workflow integration, and usability. Ethical, legal, and governance issues are only rarely addressed explicitly, with most attention focused on privacy and data protection.

Taken together, these findings suggest that cardiovascular digital twins are technically mature enough to support sophisticated, patient-specific simulations but are not yet ready for routine care. Realizing their potential for precision cardiology will require coordinated progress in standardized evaluation and reporting, rigorous clinical and external validation, user-centered and explainable design, robust data governance, and engagement with regulators and health systems. With the strengthening of these elements, digital twins may evolve from exploratory research tools into trusted, clinically integrated assets for individualized cardiovascular diagnosis, risk assessment, and treatment planning.

## Supplementary material

10.2196/78499 Multimedia Appendix 1 Search strategy.

10.2196/78499 Multimedia Appendix 2 Screened records.

10.2196/78499 Multimedia Appendix 3 Data extraction form.

10.2196/78499 Multimedia Appendix 4 Research question categories.

10.2196/78499 Multimedia Appendix 5 Mapping of raw extraction values to harmonized research question categories.

10.2196/78499 Checklist 1 PRISMA checklist.

## Abbreviations

AI: artificial intelligence
CFD: computational fluid dynamics
CT: computed tomography
CVD: cardiovascular disease
DL: deep learning
ECG: electrocardiography
FEM: finite element modeling
GDPR: General Data Protection Regulation
HIPAA: Health Insurance Portability and Accountability Act
ML: machine learning
MRI: magnetic resonance imaging
PICO: Population, Intervention, Comparison, and Outcome
PRISMA: Preferred Reporting Items for Systematic Reviews and Meta-Analyses
PROBAST: Prediction Model Risk of Bias Assessment Tool
ROBINS-I: Risk of Bias in Non-Randomized Studies - of Interventions
RQ: research question
