# Supplementary material for: Technologies, Clinical Applications, and Implementation Barriers of Digital Twins in Precision Cardiology: Systematic Review
Source: JMIR Cardio. 2026 Jan 8;10:e78499. doi: 10.2196/78499 (PMC12782626; doi:10.2196/78499)
Supplement: Multimedia Appendix 1 [file cardio-v10-e78499-s001.doc]

Multimedia Appendix 1. Search Strategy

**PubMed:**

(("digital twin*"[Title/Abstract] OR "virtual twin*"[Title/Abstract] OR "digital patient model*"[Title/Abstract] OR "virtual patient model*"[Title/Abstract] OR "in silico model*"[Title/Abstract] OR "simulation model*"[Title/Abstract] OR "cyber-physical system*"[Title/Abstract]) AND ("personalized"[Title/Abstract] AND "medicine"[Title/Abstract] OR "precision medicine"[MeSH Terms] OR "precision medicine"[Title/Abstract] OR "individualized medicine"[Title/Abstract] OR "targeted therap*"[Title/Abstract] OR "patient-specific treatment"[Title/Abstract] OR "personalized"[Other Term] OR "precision"[Other Term] OR "medicine"[Other Term]) AND ("cardiovascular diseases"[MeSH Terms] OR "heart diseases"[MeSH Terms] OR "heart*"[Title/Abstract] OR "cardiovascular*"[Title/Abstract] OR "cardiac*"[Title/Abstract] OR "aortic*"[Title/Abstract] OR "cardiology"[Title/Abstract] OR "circulatory disease"[Title/Abstract] OR "coronary artery disease"[Title/Abstract] OR "cardiomyopathy"[Title/Abstract] OR "atherosclerosis"[Title/Abstract] OR "myocardial ischemia"[Title/Abstract] OR "ischemic heart disease"[Title/Abstract] OR "heart failure"[Title/Abstract] OR "catheter ablation"[Title/Abstract] OR "aortography"[Title/Abstract] OR "myocardium"[Title/Abstract] OR "cardiology"[Other Term] OR "circulatory disease"[Other Term] OR "coronary artery disease"[Other Term] OR "cardiomyopathy"[Other Term] OR "atherosclerosis"[Other Term] OR "myocardial ischemia"[Other Term] OR "ischemic heart disease"[Other Term] OR "heart failure"[Other Term] OR "catheter ablation"[Other Term] OR "aortography"[Other Term] OR "myocardium"[Other Term]))

**IEEE:**

(("Abstract":"digital twin" OR "Title":"digital twin" OR "Abstract":"virtual twin" OR "Title":"virtual twin" OR "Abstract":"digital patient" OR "Title":"digital patient" OR "Abstract":"virtual patient" OR "Title":"virtual patient" OR "Abstract":"simulation model" OR "Title":"simulation model")

AND

("Abstract":"personalized medicine" OR "Title":"personalized medicine" OR "Abstract":"precision medicine" OR "Title":"precision medicine" OR "Abstract":"individualized medicine" OR "Title":"individualized medicine" OR "Abstract":"targeted therapy" OR "Title":"targeted therapy" OR "Abstract":"patient-specific treatment" OR "Title":"patient-specific treatment")

AND

("Abstract":"heart" OR "Title":"heart" OR "Abstract":"cardiovascular" OR "Title":"cardiovascular" OR "Abstract":"cardiac" OR "Title":"cardiac" OR "Abstract":"aortic" OR "Title":"aortic" OR "Abstract":"cardiology" OR "Title":"cardiology" OR "Abstract":"circulatory disease" OR "Title":"circulatory disease" OR "Abstract":"coronary artery disease" OR "Title":"coronary artery disease" OR "Abstract":"cardiomyopathy" OR "Title":"cardiomyopathy" OR "Abstract":"atherosclerosis" OR "Title":"atherosclerosis" OR "Abstract":"myocardial ischemia" OR "Title":"myocardial ischemia" OR "Abstract":"ischemic heart disease" OR "Title":"ischemic heart disease" OR "Abstract":"heart failure" OR "Title":"heart failure" OR "Abstract":"catheter ablation" OR "Title":"catheter ablation" OR "Abstract":"aortography" OR "Title":"aortography" OR "Abstract":"myocardium" OR "Title":"myocardium"))

**Scopus:**

TITLE-ABS-KEY(("digital twin" OR "virtual twin" OR "digital patient model" OR "virtual patient model" OR "in silico model" OR "simulation model" OR "cyber-physical system")

AND

("personalized medicine" OR "precision medicine" OR "individualized medicine" OR "targeted therapy" OR "patient-specific treatment")

AND

("heart*" OR "cardiovascular*" OR "cardiac*" OR "aortic*" OR "cardiology" OR "circulatory disease" OR "coronary artery disease" OR "cardiomyopathy" OR "atherosclerosis" OR "myocardial ischemia" OR "ischemic heart disease" OR "heart failure" OR "catheter ablation" OR "aortography" OR "myocardium"))

**WOS:**

TS=("digital twin" OR "virtual twin" OR "digital patient model" OR "virtual patient model" OR "in silico model" OR "simulation model" OR "cyber-physical system")

AND

TS=("personalized medicine" OR "precision medicine" OR "individualized medicine" OR "targeted therapy" OR "patient-specific treatment")

AND

TS=("heart*" OR "cardiovascular*" OR "cardiac*" OR "aortic*" OR "cardiology" OR "circulatory disease" OR "coronary artery disease" OR "cardiomyopathy" OR "atherosclerosis" OR "myocardial ischemia" OR "ischemic heart disease" OR "heart failure" OR "catheter ablation" OR "aortography" OR "myocardium")

**Google Scholar:**

intitle:("digital twin" OR "virtual twin" OR "digital patient model" OR "virtual patient model" OR "in silico model" OR "simulation model" OR "cyber-physical system")

("personalized medicine" OR "precision medicine" OR "individualized medicine" OR "targeted therapy" OR "patient-specific treatment")

("heart*" OR "cardiovascular*" OR "cardiac*" OR "aortic*" OR "cardiology" OR "circulatory disease" OR "coronary artery disease" OR "cardiomyopathy" OR "atherosclerosis" OR "myocardial ischemia" OR "ischemic heart disease" OR "heart failure" OR "catheter ablation" OR "aortography" OR "myocardium")
