# Supplementary material for: Technologies, Clinical Applications, and Implementation Barriers of Digital Twins in Precision Cardiology: Systematic Review
Source: JMIR Cardio. 2026 Jan 8;10:e78499. doi: 10.2196/78499 (PMC12782626; doi:10.2196/78499)
Supplement: Multimedia Appendix 2 [file cardio-v10-e78499-s002.docx]

**Multimedia Appendix 2. Screened Records**

**Filtering Questions:**

| FQ1: Does the study relate to Digital Twins in healthcare or medicine? |
| --- |
| FQ2: Does the study specifically address the use of Digital Twins in Cardiology? |
| FQ3: Does the study involve personalized or patient-specific applications in cardiology? |

| ID | Title | Year | DOI | Final Inclusion Decision | Exclude Reason |
| --- | --- | --- | --- | --- | --- |
| 1 | Digital twins as global learning health and disease models for preventive and personalized medicine. | 2025 | 10.1186/s13073-025-01435-7 | Exclude | FQ2 = No |
| 2 | Probabilistic learning of the Purkinje network from the electrocardiogram. | 2025 | 10.1016/j.media.2025.103460 | Include |  |
| 3 | Survey and perspective on verification, validation, and uncertainty quantification of digital twins for precision medicine. | 2025 | 10.1038/s41746-025-01447-y | Exclude | Publication type is not match. |
| 4 | Impact of thrombus composition on virtual thrombectomy procedures using human clot analogues mechanical data. | 2025 | 10.1016/j.jmbbm.2025.106886 | Exclude | FQ2 = No |
| 5 | Complement inhibition targets a rich-club within the neuroinflammatory network after stroke to improve radiographic and functional outcomes. | 2025 | 10.1186/s12974-024-03316-z | Exclude | FQ1 = No |
| 6 | Evolution in electrophysiology 100 years after Einthoven: translational and computational innovations in rhythm control of atrial fibrillation. | 2024 | 10.1093/europace/euae304 | Exclude | Publication type is not match. |
| 7 | From bits to bedside: entering the age of digital twins in cardiac electrophysiology. | 2024 | 10.1093/europace/euae295 | Exclude | Publication type is not match. |
| 8 | A scalable framework for learning the geometry-dependent solution operators of partial differential equations. | 2024 | 10.1038/s43588-024-00732-2 | Include |  |
| 9 | Personalized nutrition in type 2 diabetes remission: application of digital twin technology for predictive glycemic control. | 2024 | 10.3389/fendo.2024.1485464 | Exclude | FQ2 = No |
| 10 | Harnessing 12-lead ECG and MRI data to personalise repolarisation profiles in cardiac digital twin models for enhanced virtual drug testing. | 2025 | 10.1016/j.media.2024.103361 | Include |  |
| 11 | Definitions and Characteristics of Patient Digital Twins Being Developed for Clinical Use: Scoping Review. | 2024 | 10.2196/58504 | Exclude | Publication type is not match. |
| 12 | Defining myocardial fiber bundle architecture in atrial digital twins. | 2024 |  | Include |  |
| 13 | Cardiovascular care with digital twin technology in the era of generative artificial intelligence. | 2024 | 10.1093/eurheartj/ehae619 | Exclude | Publication type is not match. |
| 14 | Design of a Digital Twin of the Heart for the Management of Heart Failure Patients. | 2024 | 10.3233/SHTI240551 | Include |  |
| 15 | [Healthcare 4.0-Medicine in transition]. | 2024 | 10.1007/s00059-024-05267-w | Exclude |  |
| 16 | Building Digital Twins for Cardiovascular Health: From Principles to Clinical Impact. | 2024 | 10.1161/JAHA.123.031981 | Exclude | Publication type is not match. |
| 17 | Patient-specific 3D coronary model in cardiac catheterisation laboratories. | 2024 | 10.3389/fcvm.2024.1398290 | Exclude | Publication type is not match. |
| 18 | Fluid-structure interaction simulation of mechanical aortic valves: a narrative review exploring its role in total product life cycle. | 2024 | 10.3389/fmedt.2024.1399729 | Exclude | FQ3 = No |
| 19 | Computational modeling of heart failure in microgravity transitions. | 2024 | 10.3389/fphys.2024.1351985 | Exclude | FQ3 = No |
| 20 | Personalized evaluation of the passive myocardium in ischemic cardiomyopathy via computational modeling using Bayesian optimization. | 2024 | 10.1007/s10237-024-01856-0 | Include |  |
| 21 | Digital twins: reimagining the future of cardiovascular risk prediction and personalised care. | 2024 | 10.1016/j.hjc.2024.06.001 | Exclude |  |
| 22 | Digital twins for cardiac electrophysiology: state of the art and future challenges. | 2024 | 10.1007/s00399-024-01014-0 | Exclude | Publication type is not match. |
| 23 | Whole-heart electromechanical simulations using Latent Neural Ordinary Differential Equations. | 2024 | 10.1038/s41746-024-01084-x | Include |  |
| 24 | Digital twins in medicine. | 2024 | 10.1038/s43588-024-00607-6 | Exclude | Publication type is not match. |
| 25 | Metabolic profiling of aortic stenosis and hypertrophic cardiomyopathy identifies mechanistic contrasts in substrate utilization. | 2024 | 10.1096/fj.202301710RR | Exclude | FQ1 = No |
| 26 | Grand Challenges at the Interface of Engineering and Medicine. | 2024 | 10.1109/OJEMB.2024.3351717 | Exclude | FQ2 = No |
| 27 | A computational model predicts sex-specific responses to calcium channel blockers in mammalian mesenteric vascular smooth muscle. | 2024 | 10.7554/eLife.90604 | Exclude | FQ3 = No |
| 28 | Integrating evidence and causal mapping of factors that influence medication decision-making by pregnant women at risk of hypertensive disorder: protocol for a scoping review. | 2024 | 10.1136/bmjopen-2023-074775 | Exclude | FQ1 = No |
| 29 | Digitalomics, digital intervention, and designing future: The next frontier in cardiology. | 2024 | 10.1016/j.jjcc.2023.12.002 | Exclude | Publication type is not match. |
| 30 | Toward Digital Twin Technology for Precision Pharmacology. | 2024 | 10.1016/j.jacep.2023.10.024 | Include |  |
| 31 | Digital Twinning of Cardiac Electrophysiology Models From the Surface ECG: A Geodesic Backpropagation Approach. | 2024 | 10.1109/TBME.2023.3331876 | Include |  |
| 32 | Mechanical modeling of the maturation process for tissue-engineered implants: Application to biohybrid heart valves. | 2023 | 10.1016/j.compbiomed.2023.107623 | Exclude | FQ3 = No |
| 33 | Digital Twins in Healthcare: Methodological Challenges and Opportunities. | 2023 | 10.3390/jpm13101522 | Exclude | Publication type is not match. |
| 34 | Up digital and personal: How heart digital twins can transform heart patient care. | 2024 | 10.1016/j.hrthm.2023.10.019 | Exclude | Publication type is not match. |
| 35 | Computational Biomedicine (CompBioMed) Centre of Excellence: Selected Key Achievements. | 2024 | 10.1007/978-1-0716-3449-3_3 | Exclude | FQ2 = No |
| 36 | Inhibitor of DNA Binding Protein 3 (ID3) and Nuclear Respiratory Factor 1 (NRF1) Mediated Transcriptional Gene Signatures are Associated with the Severity of Cerebral Amyloid Angiopathy. | 2024 | 10.1007/s12035-023-03541-2 | Exclude | FQ1 = No |
| 37 | Wearable Technologies and AI at the Far Edge for Chronic Heart Failure Prevention and Management: A Systematic Review and Prospects. | 2023 | 10.3390/s23156896 | Exclude | FQ1 = No |
| 38 | Using the Non-Adoption, Abandonment, Scale-Up, Spread, and Sustainability (NASSS) Framework to Identify Barriers and Facilitators for the Implementation of Digital Twins in Cardiovascular Medicine. | 2023 | 10.3390/s23146333 | Exclude | Publication type is not match. |
| 39 | Editorial: Computational methods in cardiac electrophysiology. | 2023 | 10.3389/fphys.2023.1231342 | Exclude |  |
| 40 | Mapping the use of computational modelling and simulation in clinics: A survey. | 2023 | 10.3389/fmedt.2023.1125524 | Exclude | FQ2 = No |
| 41 | Digital twins and the future of precision mental health. | 2023 | 10.3389/fpsyt.2023.1082598 | Exclude | FQ2 = No |
| 42 | Putting the Personalized Metabolic Avatar into Production: A Comparison between Deep-Learning and Statistical Models for Weight Prediction. | 2023 | 10.3390/nu15051199 | Exclude | FQ2 = No |
| 43 | Mechanism based therapies enable personalised treatment of hypertrophic cardiomyopathy. | 2022 | 10.1038/s41598-022-26889-2 | Include |  |
| 44 | In silico model of atherosclerosis with individual patient calibration to enable precision medicine for cardiovascular disease. | 2023 | 10.1016/j.compbiomed.2022.106364 | Include |  |
| 45 | A personalized 0D-1D model of cardiovascular system for the hemodynamic simulation of enhanced external counterpulsation. | 2022 | 10.1016/j.cmpb.2022.107224 | Include |  |
| 46 | The health digital twin to tackle cardiovascular disease-a review of an emerging interdisciplinary field. | 2022 | 10.1038/s41746-022-00640-7 | Exclude | Publication type is not match. |
| 47 | Personalized ablation vs. conventional ablation strategies to terminate atrial fibrillation and prevent recurrence. | 2023 | 10.1093/europace/euac116 | Include |  |
| 48 | Building digital twins of the human immune system: toward a roadmap. | 2022 | 10.1038/s41746-022-00610-z | Exclude | FQ2 = No |
| 49 | An Integrated Workflow for Building Digital Twins of Cardiac Electromechanics-A Multi-Fidelity Approach for Personalising Active Mechanics. | 2022 | 10.3390/math10050823 | Include |  |
| 50 | Medicine-Based Evidence in Congenital Heart Disease: How Artificial Intelligence Can Guide Treatment Decisions for Individual Patients. | 2021 | 10.3389/fcvm.2021.798215 | Exclude | Publication type is not match. |
| 51 | Multiscale Computational Modeling of Vascular Adaptation: A Systems Biology Approach Using Agent-Based Models. | 2021 | 10.3389/fbioe.2021.744560 | Exclude | FQ1 = No |
| 52 | A completely automated pipeline for 3D reconstruction of human heart from 2D cine magnetic resonance slices. | 2021 | 10.1098/rsta.2020.0257 | Include |  |
| 53 | The health digital twin: advancing precision cardiovascular medicine. | 2021 | 10.1038/s41569-021-00630-4 | Exclude |  |
| 54 | Automated Framework for the Inclusion of a His-Purkinje System in Cardiac Digital Twins of Ventricular Electrophysiology. | 2021 | 10.1007/s10439-021-02825-9 | Include |  |
| 55 | Arrhythmogenic Effects of Genetic Mutations Affecting Potassium Channels in Human Atrial Fibrillation: A Simulation Study. | 2021 | 10.3389/fphys.2021.681943 | Exclude | FQ3 = No |
| 56 | A precision medicine framework for personalized simulation of hemodynamics in cerebrovascular disease. | 2021 | 10.1186/s12938-021-00880-w | Exclude | FQ2 = No |
| 57 | Configuration of vascular services: a multiple methods research programme |  |  | Exclude |  |
| 58 | Precision Medicine and cardiac channelopathies: when dreams meet reality. | 2021 | 10.1093/eurheartj/ehab007 | Exclude | Publication type is not match. |
| 59 | Cost-utility analysis of an integrated genetic/epigenetic test for assessing risk for coronary heart disease. | 2021 | 10.2217/epi-2021-0021 | Exclude | FQ1 = No |
| 60 | Precision medicine in human heart modeling : Perspectives, challenges, and opportunities. | 2021 | 10.1007/s10237-021-01421-z | Exclude | Publication type is not match. |
| 61 | Prioritized Research for the Prevention, Treatment, and Reversal of Chronic Disease: Recommendations From the Lifestyle Medicine Research Summit. | 2020 | 10.3389/fmed.2020.585744 | Exclude | FQ1 = No |
| 62 | Review of simulation model for education of point-of-care ultrasound using easy-to-make tools. | 2020 | 10.12998/wjcc.v8.i19.4286 | Exclude | FQ1 = No |
| 63 | Using Digital Twins for Precision Medicine in Vascular Surgery. | 2020 | 10.1016/j.avsg.2020.04.042 | Exclude |  |
| 64 | The 'Digital Twin' to enable the vision of precision cardiology. | 2020 | 10.1093/eurheartj/ehaa159 | Exclude | Publication type is not match. |
| 65 | Rationale and design for studying organisation of care for intra-arterial thrombectomy in the Netherlands: simulation modelling study. | 2020 | 10.1136/bmjopen-2019-032754 | Exclude | FQ1 = No |
| 66 | Cardiovascular models for personalised medicine: Where now and where next? | 2019 | 10.1016/j.medengphy.2019.08.007 | Exclude | Publication type is not match. |
| 67 | Evaluation of emotional excitation during standardized endotracheal intubation in simulated conditions. | 2018 | 10.1186/s13613-018-0460-0 | Exclude | FQ1 = No |
| 68 | A Cost-Effective, Rapidly Constructed Simulation Model for Ultrasound-Guided Pericardiocentesis Procedural Training. | 2019 | 10.1016/j.jemermed.2018.09.010 | Exclude | FQ1 = No |
| 69 | [Computer-assisted decision-making in cardiac surgery: from 3D preoperative planning to computational fluid dynamics in the design of surgical procedures]. | 2018 | 10.1556/1046.71.2018.3.2 | Exclude |  |
| 70 | Large-scale in silico identification of drugs exerting sex-specific effects in the heart. | 2018 | 10.1186/s12967-018-1612-6 | Exclude | FQ3 = No |
| 71 | Real Patient and its Virtual Twin: Application of Quantitative Systems Toxicology Modelling in the Cardiac Safety Assessment of Citalopram. | 2017 | 10.1208/s12248-017-0155-8 | Include |  |
| 72 | A Novel Low-Cost Ultrasound-Guided Pericardiocentesis Simulation Model: Demonstration of Feasibility. | 2018 | 10.1002/jum.14337 | Exclude | FQ1 = No |
| 73 | Cost-effectiveness modelling of telehealth for patients with raised cardiovascular disease risk: evidence from a cohort simulation conducted alongside the Healthlines randomised controlled trial. | 2016 | 10.1136/bmjopen-2016-012355 | Exclude | FQ1 = No |
| 74 | Integrated whole-heart computational workflow for inverse potential mapping and personalized simulations. | 2016 | 10.1186/s12967-016-0902-0 | Include |  |
| 75 | Intubation after rapid sequence induction performed by non-medical personnel during space exploration missions: a simulation pilot study in a Mars analogue environment. | 2015 | 10.1186/s13728-015-0038-5 | Exclude | FQ2 = No |
| 76 | Aortic dissection simulation models for clinical support: fluid-structure interaction vs. rigid wall models. | 2015 | 10.1186/s12938-015-0032-6 | Include |  |
| 77 | Towards personalized clinical in-silico modeling of atrial anatomy and electrophysiology. | 2013 | 10.1007/s11517-012-0970-0 | Exclude | Publication type is not match. |
| 78 | SNP analysis of Rac1 For personalized ligand interaction. | 2010 | 10.1109/IEMBS.2010.5626750 | Exclude | FQ1 = No |
| 79 | [In-silico models for the simulation and prediction of the cardiac function]. | 2010 | 10.1016/S0929-693X(10)70022-1 | Exclude |  |
| 80 | Cardiac Healthcare Digital Twins Supported by Artificial Intelligence-Based Algorithms and Extended Reality-A Systematic Review | 2024 | 10.3390/electronics13050866 | Exclude | Publication type is not match. |
| 81 | Digital twin in healthcare: Recent updates and challenges | 2023 | 10.1177/20552076221149651 | Exclude | FQ2 = No |
| 82 | How to Address Uncertainty in Health Economic Discrete-Event Simulation Models: An Illustration for Chronic Obstructive Pulmonary Disease | 2020 | 10.1177/0272989X20932145 | Exclude | FQ1 = No |
| 83 | Electro-Mechanical Whole-Heart Digital Twins: A Fully Coupled Multi-Physics Approach | 2021 | 10.3390/math9111247 | Include |  |
| 84 | Current state-of-the-art and utilities of machine learning for detection, monitoring, growth prediction, rupture risk assessment, and post-surgical management of abdominal aortic aneurysms | 2022 | 10.1016/j.apples.2022.100097 | Exclude | Publication type is not match. |
| 85 | Collective Variational Inference for Personalized and Generative Physiological Modeling: A Case Study on Hemorrhage Resuscitation | 2022 | 10.1109/TBME.2021.3103141 | Exclude | FQ2 = No |
| 86 | Increasing acceptance of AI-generated digital twins through clinical trial applications | 2024 | 10.1111/cts.13897 | Exclude | FQ2 = No |
| 87 | Nexus between in silico and in vivo models to enhance clinical translation of nanomedicine | 2021 | 10.1016/j.nantod.2020.101057 | Exclude | FQ2 = No |
| 88 | Opportunities and challenges of digital twin technology in healthcare | 2023 | 10.1097/CM9.0000000000002896 | Exclude |  |
| 89 | Computer Simulation Model May Prevent Thoracic Stent-Graft Collapse Complication | 2022 | 10.1161/CIRCIMAGING.121.013764 | Exclude |  |
| 90 | Enhanced approaches in decision support system using ai for achieving precision medicine | 2020 |  | Exclude | FQ1 = No |
| 91 | The digital twin in endovascular repair | 2019 | 10.1007/s00772-019-00569-4 | Exclude |  |
| 92 | Is personal physiology-based rapid prediction digital twin for minimal effective fentanyl dose better than standard practice: a pilot study protocol | 2024 | 10.1136/bmjopen-2024-085296 | Exclude | FQ2 = No |
| 93 | Digital Twins for Multiple Sclerosis | 2021 | 10.3389/fimmu.2021.669811 | Exclude | FQ2 = No |
| 94 | On-chip network-enabled many-core architectures for computational biology applications | 2015 | 10.7873/date.2015.1128 | Exclude | FQ1 = No |
| 95 | A Comprehensive Survey on Enhancing Patient Care Through Deep Learning and IoT-Enabled Healthcare Innovations | 2025 | 10.1007/978-981-97-8031-0_25 | Exclude | FQ1 = No |
| 96 | Building A Pipeline for Precision Antiarrhythmic Therapy | 2024 | 10.1016/j.jacep.2023.11.016 | Exclude |  |
| 97 | Digital twins in healthcare: Applications, technologies, simulations, and future trends | 2024 | 10.1002/widm.1559 | Exclude | Publication type is not match. |
| 98 | Precision Nomothetic Medicine in Depression Research: A New Depression Model, and New Endophenotype Classes and Pathway Phenotypes, and A Digital Self | 2022 | 10.3390/jpm12030403 | Exclude | FQ2 = No |
| 99 | Cost-effectiveness of a stepwise cardiometabolic disease prevention program: results of a randomized controlled trial in primary care | 2021 | 10.1186/s12916-021-01933-6 | Exclude | FQ1 = No |
| 100 | Sensitivity analysis of closed-loop one-chamber and four-chamber models with baroreflex | 2024 | 10.1371/journal.pcbi.1012377 | Exclude | FQ3 = No |
| 101 | Personalised simulation of hemodynamics in cerebrovascular disease: lessons learned from a study of diagnostic accuracy | 2023 | 10.3389/fneur.2023.1230402 | Exclude | FQ2 = No |
| 102 | Sex matters: the frequently overlooked importance of considering sex in computational models | 2023 | 10.3389/fphys.2023.1186646 | Exclude | Publication type is not match. |
| 103 | In Silico Pharmacoepidemiologic Evaluation of Drug-Induced Cardiovascular Complications Using Combined Classifiers | 2018 | 10.1021/acs.jcim.7b00641 | Exclude | FQ1 = No |
| 104 | Graph Representation Forecasting of Patient's Medical Conditions: Toward a Digital Twin | 2021 | 10.3389/fgene.2021.652907 | Exclude | FQ2 = No |
| 105 | Computational modelling for congenital heart disease: How far are we from clinical translation? | 2017 | 10.1136/heartjnl-2016-310423 | Exclude | Publication type is not match. |
| 106 | AutoGenHR: Automated Generation of Health Reports for Patients at Home | 2024 |  | Exclude | FQ1 = No |
| 107 | Electrophysiological and calcium-handling development during long-term culture of human-induced pluripotent stem cell-derived cardiomyocytes | 2023 | 10.1007/s00395-022-00973-0 | Exclude | FQ3 = No |
| 108 | Updating and calibrating the Real-World Progression In Diabetes (RAPIDS) model in a non-Veterans Affairs population | 2024 | 10.1111/dom.15878 | Exclude | FQ2 = No |
| 109 | An In Silico Model for Predicting the Efficacy of Edge-to-Edge Repair for Mitral Regurgitation | 2024 | 10.1115/1.4064055 | Include |  |
| 110 | Digital twin | 2023 | 10.1016/B978-0-12-824010-6.00051-4 | Exclude |  |
| 111 | Avoiding big data pitfalls | 2020 |  | Exclude | Publication type is not match. |
| 112 | Cyber-Physical Platform for Preeclampsia Detection | 2020 | 10.1007/978-3-030-58814-4_48 | Exclude | FQ1 = No |
| 113 | Digital twin in cardiology: Navigating the digital landscape for education, global health, and preventive medicine | 2024 | 10.1016/B978-0-443-13619-1.00006-4 | Exclude |  |
| 114 | From transcriptomics to digital twins of organ function | 2024 | 10.3389/fcell.2024.1240384 | Exclude | FQ2 = No |
| 115 | Next-generation, personalised, model-based critical care medicine: A state-of-the art review of in silico virtual patient models, methods, and cohorts, and how to validation them | 2018 | 10.1186/s12938-018-0455-y | Exclude | FQ2 = No |
| 116 | Mavacamten Efficacy in Mutation-specific Hypertrophic Cardiomyopathy: An in Silico Approach to Inform Precision Medicine | 2021 | 10.23919/CinC53138.2021.9662736 | Include |  |
| 117 | Artificial Intelligence and Health Inequities in Dietary Interventions on Atherosclerosis: A Narrative Review | 2024 | 10.3390/nu16162601 | Exclude | Publication type is not match. |
| 118 | Digital Twins in Healthcare: An Architectural Proposal and Its Application in a Social Distancing Case Study | 2023 | 10.1109/JBHI.2022.3205506 | Exclude | FQ2 = No |
| 119 | New Era of Measurable Surgical Risk Predictor by 3D Quantitative CT on Pulmonary Venous Return | 2024 | 10.1016/j.jacasi.2024.06.001 | Exclude |  |
| 120 | Integrating sex and gender in model simulations of cardiovascular flows: a narrative review | 2023 | 10.1723/4031.40065 | Exclude | FQ3 = No |
| 121 | Focus on hypertension but also on the 'the digital twin' and on kidney function and disease | 2020 | 10.1093/eurheartj/ehaa1014 | Exclude |  |
| 122 | Digital twins in healthcare and biomedicine | 2023 | 10.1016/B978-0-443-21598-8.00011-7 | Exclude |  |
| 123 | Enhancing Cardiac Health: Digital Twin Technology for Real- Time Monitoring and Abnormality Detection of Human Heart | 2024 | 10.1109/MLISE62164.2024.10674146 | Include |  |
| 124 | Multi-scale, tailor-made heart simulation can predict the effect of cardiac resynchronization therapy | 2017 | 10.1016/j.yjmcc.2017.05.006 | Include |  |
| 125 | A Review of AI-Driven Digital Twin Frameworks for Cardiovascular Disease Diagnosis and Management | 2024 | 10.1109/ITMS64072.2024.10741948 | Exclude | Publication type is not match. |
| 126 | Digital Twin Technology: The Future of Predicting Neurological Complications of Pediatric Cancers and Their Treatment | 2022 | 10.3389/fonc.2021.781499 | Exclude | FQ2 = No |
| 127 | Digital Twin for Healthcare and Lifesciences | 2023 | 10.1007/978-3-031-21343-4_32 | Exclude |  |
| 128 | The role of artificial intelligence in hypertensive disorders of pregnancy: towards personalized healthcare | 2023 | 10.1080/14779072.2023.2223978 | Exclude | FQ1 = No |
| 129 | Optimizing the Distribution of Ablation Lesions to Prevent Postablation Atrial Tachycardia: A Personalized Digital-Twin Study | 2024 | 10.1016/j.jacep.2024.07.002 | Include |  |
| 130 | Credibility assessment of patient-specific computational modeling using patient-specific cardiac modeling as an exemplar | 2022 | 10.1371/journal.pcbi.1010541 | Include |  |
| 131 | Prediction of lung mechanics throughout recruitment maneuvers in pressure-controlled ventilation | 2020 | 10.1016/j.cmpb.2020.105696 | Exclude | FQ2 = No |
| 132 | The future of precision diabetes: Digital twin | 2024 | 10.1016/B978-0-323-98808-7.00021-7 | Exclude |  |
| 133 | Healthcare 5.0: A Study on Improving Personalized Care | 2022 | 10.1109/ICICCS53718.2022.9788411 | Exclude | FQ1 = No |
| 134 | Editorial: Fractal and Multifractal Facets in the Structure and Dynamics of Physiological Systems and Applications to Homeostatic Control, Disease Diagnosis and Integrated Cyber-Physical Platforms | 2020 | 10.3389/fphys.2020.00447 | Exclude |  |
| 135 | Digital patient twins for personalized therapeutics and pharmaceutical manufacturing | 2023 | 10.3389/fdgth.2023.1302338 | Exclude | FQ2 = No |
| 136 | Deep Computational Model for the Inference of Ventricular Activation Properties | 2022 | 10.1007/978-3-031-23443-9_34 | Include |  |
| 137 | A Simulation Model to Evaluate the Implications of Genetic Testing in Cholesterol Treatment Plans | 2019 | 10.1109/WSC40007.2019.9004735 | Exclude | FQ1 = No |
| 138 | Editorial: Diagnosis, monitoring, and treatment of heart rhythm: new insights and novel computational methods | 2023 | 10.3389/fphys.2023.1272377 | Exclude |  |
| 139 | Intelligent Digital Twins for Personalized Migraine Care | 2023 | 10.3390/jpm13081255 | Exclude | FQ2 = No |
| 140 | Computational modelling of biological systems: Tools and visions | 2000 | 10.1098/rsta.2000.0547 | Exclude | Publication type is not match. |
| 141 | Generation of histo-anatomically representative models of the individual heart: Tools and application | 2009 | 10.1098/rsta.2009.0056 | Include |  |
| 142 | Personalization of a Hemodynamic Cardiac Digital Twin: An Echocardiogram based Approach | 2024 | 10.1109/BIBM62325.2024.10822244 | Include |  |
| 143 | Leveraging Digital Twin Technology to Combat Cardiovascular Disease: A Comprehensive Review | 2024 | 10.1109/IDICAIEI61867.2024.10842856 | Exclude |  |
| 144 | The Digital Twin: Modular Model-Based Approach to Personalized Medicine | 2021 | 10.1515/cdbme-2021-2057 | Exclude | FQ2 = No |
| 145 | TWIN: Personalized Clinical Trial Digital Twin Generation | 2023 | 10.1145/3580305.3599534 | Exclude |  |
| 146 | The digital twin in medicine: a key to the future of healthcare? | 2022 |  | Exclude |  |
| 147 | Digital twins: the new frontier for personalized medicine? | 2023 |  | Exclude |  |
| 148 | Personal digital twin: a close look into the present and a step towards the future of personalised healthcare industry | 2022 |  | Exclude |  |
| 149 | Digital Twins for Predictive, Preventive Personalized, and Participatory Treatment of Immune-Mediated Diseases | 2023 | 10.1161/ATVBAHA.122.318331 | Exclude | FQ2 = No |
| 150 | A multidisciplinary hyper-modeling scheme in personalized in silico oncology: coupling cell kinetics with metabolism, signaling networks, and biomechanics as plug-in component models of a cancer digital twin | 2024 |  | Exclude |  |
| 151 | Towards continuous monitoring in personalized healthcare through digital twins | 2019 |  | Exclude |  |
| 152 | Dynamic digital twin: Diagnosis, treatment, prediction, and prevention of disease during the life course | 2022 |  | Exclude |  |
| 153 | The health digital twin to tackle cardiovascular disease—a review of an emerging interdisciplinary field | 2022 |  | Exclude |  |
| 154 | Digital Twin Technology and Applications in the Medical Field: Bridging the Virtual for Precision Medicine | 2024 |  | Exclude |  |
| 155 | Digital twins: from personalised medicine to precision public health | 2021 |  | Exclude |  |
| 156 | Toward precision medicine using a “digital twin” approach: modeling the onset of disease-specific brain atrophy in individuals with multiple sclerosis | 2023 |  | Exclude |  |
| 157 | Digital Twin Disease Diagnosis Using Machine Learning | 2021 |  | Exclude |  |
| 158 | The ‘Digital Twin’to enable the vision of precision cardiology | 2020 |  | Exclude |  |
| 159 | Dynamic mirroring: unveiling the role of digital twins, artificial intelligence and synthetic data for personalized medicine in laboratory medicine | 2024 | 10.1515/cclm-2024-0517 | Exclude | FQ2 = No |
| 160 | Graph representation forecasting of patient's medical conditions: Toward a digital twin | 2021 |  | Exclude |  |
| 161 | Networking architecture and key supporting technologies for human digital twin in personalized healthcare: A comprehensive survey | 2023 |  | Exclude |  |
| 162 | The digital twin revolution in healthcare | 2020 |  | Exclude |  |
| 163 | Current Progress of Digital Twin Construction Using Medical Imaging | 2024 | 10.48550/arXiv.2411.08173 | Exclude |  |
| 164 | Digital twin technology: revolutionary to improve personalized healthcare | 2020 |  | Exclude |  |
| 165 | Digital twin for healthcare systems | 2023 |  | Exclude |  |
| 166 | The digital twin: A potential solution for the personalized diagnosis and treatment of musculoskeletal system diseases | 2023 |  | Exclude |  |
| 167 | Human Digital Twin Processes and their Future | 2024 |  | Exclude |  |
| 168 | A perfectly imperfect engine: Utilizing the digital twin paradigm in pulmonary hypertension | 2024 | 10.1002/pul2.12392 | Exclude | Publication type is not match. |
| 169 | Design of precision medicine web-service platform towards health care digital twin | 2023 |  | Exclude |  |
| 170 | Medical Digital Twin: A Review on Technical Principles and Clinical Applications | 2025 |  | Exclude |  |
| 171 | Human digital twin for personalized elderly type 2 diabetes management | 2023 |  | Exclude |  |
| 172 | Digital-Twin-Enabled IoMT system for surgical simulation using rAC-GAN | 2022 |  | Exclude |  |
| 173 | Virtual twin for healthcare management | 2023 |  | Exclude |  |
| 174 | Digital Twin Ecosystem for Oncology Clinical Operations | 2024 | 10.48550/arXiv.2409.17650 | Exclude |  |
| 175 | A multidisciplinary approach to the development of digital twin models of critical care delivery in intensive care units | 2022 | 10.1080/00207543.2021.2022235 | Exclude |  |
| 176 | Digital Twin in Health Care | 2023 |  | Exclude |  |
| 177 | Digital Twin Generators for Disease Modeling | 2024 | 10.48550/arXiv.2405.01488 | Exclude |  |
| 178 | A Proposed Framework for Digital Twins Driven Precision Medicine Platform: Values and Challenges | 2022 |  | Exclude |  |
| 179 | From evidence-based medicine to digital twin technology for predicting ventricular tachycardia in ischaemic cardiomyopathy | 2022 | 10.1098/rsif.2022.0317 | Exclude | Publication type is not match. |
| 180 | Digital Twin for Healthcare and Lifesciences | 2023 |  | Exclude |  |
| 181 | Human Body Digital Twin: A Master Plan | 2024 | 10.1038/s44287-024-00025-w | Exclude | FQ2 = No |
| 182 | The Role of Digital Twins in Personalized Sleep Medicine | 2022 |  | Exclude |  |
| 183 | Cardiac Digital Twin Modeling | 2022 |  | Exclude |  |
| 184 | Twinning is Winning: As disease-related data increase, digital twin technology promises to impact precision medicine from clinical trials to clinical care—in silico | 2022 | 10.1089/ipm.09.05.07 | Exclude |  |
| 185 | Evolution of Simulation and Digital Twin in Health Care: From Discovery to Design and Integration | 2024 |  | Exclude |  |
| 186 | Cardiac Digital Twin Pipeline for Virtual Therapy Evaluation | 2024 | 10.48550/arXiv.2401.10029 | Include |  |
| 187 | Hybrid disease prediction approach leveraging digital twin and metaverse technologies for health consumer | 2024 | 10.1186/s12911-024-02495-2 | Exclude | FQ2 = No |
| 188 | Immune Digital Twin Blueprint: A Comprehensive Mechanistic Model of the Human Immune System | 2020 | 10.1101/2020.03.11.988238 | Exclude |  |
| 189 | A Review of AI-Driven Digital Twin Frameworks for Cardiovascular Disease Diagnosis and Management | 2024 |  | Exclude |  |
| 190 | Application and development prospect of digital twin in the forensic identification of cardiovascular diseases | 2024 |  | Exclude | Publication type is not match. |
| 191 | Personalization of a Hemodynamic Cardiac Digital Twin: An Echocardiogram based Approach | 2024 |  | Exclude |  |
| 192 | Cardiovascular care with digital twin technology in the era of generative artificial intelligence | 2024 |  | Exclude |  |
| 193 | Digital twin in cardiology: Navigating the digital landscape for education, global health, and preventive medicine | 2025 |  | Exclude |  |
| 194 | Advancing Congestive Heart Failure Management through Digital Twin Integration |  |  | Include |  |
| 195 | Proposal of statistical twin as a transition to full digital twin technology for cardiovascular interventions | 2024 | 10.1093/icvts/ivae032/7619106 | Include |  |
| 196 | Advancing Treatment and Management of Congestive Heart Failure through Integration of Digital Twin Technology and Big Data Analytics | 2024 |  | Exclude | Publication type is not match. |
| 197 | Case study: digital twin in cardiology | 2023 |  | Exclude |  |
| 198 | Enhancing Cardiac Health: Digital Twin Technology for Real-Time Monitoring and Abnormality Detection of Human Heart | 2024 |  | Exclude |  |
| 199 | An insight in the future of healthcare: integrating digital twin for personalized medicine | 2024 | 10.1007/s12553-024-00869-0 | Exclude | FQ1 = No |
| 200 | A digital twin enabled wearable device for customized healthcare | 2025 |  | Exclude | FQ2 = No |
| 201 | Digital Twin For A Human Heart Using Deep Learning and Stream Processing Platforms | 2023 |  | Exclude |  |
| 202 | 8 The Digital Twin |  |  | Include |  |
| 203 | Digital twin for cardiology | 2023 |  | Exclude | Publication type is not match. |
| 204 | Clinical usefulness of digital twin guided virtual amiodarone test in patients with atrial fibrillation ablation | 2024 |  | Include |  |
| 205 | Towards a Design Ecosystem for a Personal Digital Twin for Well-Being | 2024 |  | Exclude |  |
| 206 | Focus on hypertension but also on the 'the digital twin'and on kidney function and disease | 2020 |  | Exclude |  |
| 207 | Revolutionizing Hypertension Management in Type 2 Diabetes: The Promise of Digital Twin Technology | 2024 | 10.1016/j.jacadv.2024.101173 | Exclude | Publication type is not match. |
| 208 | The future of precision diabetes: Digital twin | 2025 |  | Exclude |  |
| 209 | A Digital Twin-Based Platform for Medical Cyber-Physical Systems | 2024 |  | Include |  |
| 210 | The potential of the Medical Digital Twin in diabetes management: a review | 2023 | 10.3389/fmed.2023.1178912 | Exclude | FQ2 = No |
| 211 | The perioperative human digital twin | 2022 |  | Exclude | FQ2 = No |
| 212 | Integrating deep learning and reinforcement learning into a digital twin architecture for medical predictions. | 2024 |  | Include |  |
| 213 | A digital twin approach for stroke risk assessment in Atrial Fibrillation Patients | 2024 |  | Include |  |
| 214 | A Novel Medical Cyber-Physical Systems Based on Digital Twin-Driven Platform | 2024 | 10.1142/S0129156425401299 | Exclude | can`t download the paper |
| 215 | A Comparative Study on Cloud-based and Edge-Based Digital Twin Frameworks for Prediction of Cardiovascular Disease. | 2023 |  | Include |  |
| 216 | A comprehensive review of digital twin in healthcare in the scope of simulative health-monitoring | 2025 | 10.1177/20552076241304078 | Exclude | Publication type is not match. |
| 217 | … in silico oncology: coupling cell kinetics with metabolism, signaling networks, and biomechanics as plug-in component models of a cancer digital twin | 2024 |  | Exclude | FQ2 = No |
| 218 | Digital Twin Technology in Resolving Polycystic Ovary Syndrome and Improving Metabolic Health: A Comprehensive Case Study | 2025 |  | Exclude | FQ2 = No |
| 219 | A multiscale predictive digital twin for neurocardiac modulation | 2023 | 10.1113/JP284391 | Include |  |
| 220 | Leveraging the benefits of digital twin in delivering personalized medicine using IOT | 2024 |  | Exclude | FQ2 = No |
| 221 | Human digital twin technology for individual profiling using LC-MS untargeted metabolomics analysis of dried blood spot samples | 2024 |  | Exclude | FQ2 = No |
| 222 | Quantifying variabilities in cardiac digital twin models of the electrocardiogram | 2024 |  | Include |  |
| 223 | Wearable Sensor and Digital Twin Technology for the Development of a Personalized Digital Biomarker of Vaccine-Induced Inflammation | 2024 | 10.1101/2024.01.28.24301887.abstract | Exclude | FQ2 = No |
| 224 | Digital Twin Types and Applications in Healthcare | 2024 | 10.3233/SHTI241056 | Exclude | Publication type is not match. |
| 225 | Potential applications of digital twin in medical care | 2023 |  | Exclude | FQ2 = No |
| 226 | Creation of MLPA: A Multi-level Digital Twin Framework for Personalized Cancer Simulation and Treatment Optimization | 2024 | 10.1101/2024.09.13.612988.abstract | Exclude | FQ2 = No |
| 227 | Assessing Post-TAVR Cardiac Conduction Abnormalities Risk Using a Digital Twin of a Beating Heart | 2024 | 10.1101/2024.03.28.24305028.abstract | Exclude | FQ3 = No |
| 228 | An individualized digital twin of a patient for transdermal fentanyl therapy for chronic pain management | 2023 | 10.1007/s13346-023-01305-y | Exclude | FQ2 = No |
| 229 | A smart IoT platform for oncology patient diagnosis based on ai: Towards the human digital twin | 2021 |  | Exclude | FQ1 = No |
| 230 | What does a Heart Beat for? A Heterogeneous Approach for Human Digital Twin Construction | 2024 |  | Include |  |
| 231 | A digital twin model for evidence-based clinical decision support in multiple myeloma treatment | 2023 | 10.3389/fdgth.2023.1324453 | Exclude | FQ2 = No |
| 232 | Transforming Healthcare: Exploring the Impact and Potential of Digital Twin Technology | 2023 |  | Exclude | Publication type is not match. |
| 233 | Blockchain-based digital twin to predict heart attacks | 2024 | 10.1201/9781003408246-16 | Exclude | can`t download the paper |
| 234 | DECIDE-Twin: A Framework for AI-Enabled Digital Twins in Clinical Decision-Making | 2024 |  | Exclude | FQ2 = No |
| 235 | Spatiotemporal Stability of Dominant Frequency Sites in Atrial Fibrillation Using Digital Twin Technology and Clinical Outcomes of Catheter Ablation | 2024 | 10.1161/circ.150.suppl_1.4146041 | Exclude | Publication type is not match. |
| 236 | Digital Twin: Implementation | 2024 | 10.1007/978-3-031-76564-3_7 | Exclude | Publication type is not match. |
| 237 | Predictive digital twin for optimizing patient-specific radiotherapy regimens under uncertainty in high-grade gliomas | 2023 | 10.3389/frai.2023.1222612 | Exclude | FQ2 = No |
| 238 | Integrating Digital Twin Technology with Dynamic Ensemble Learning for Sepsis Prediction in Intensive Care Units | 2024 |  | Exclude | FQ2 = No |
| 239 | A multimodal model in the prediction of the delivery mode using data from a digital twin-empowered labor monitoring system | 2024 | 10.1177/20552076241304934 | Exclude | FQ2 = No |
| 240 | Engineering a Digital Twin for Diagnosis and Treatment of Multiple Sclerosis | 2024 | 10.1145/3652620.3688249 | Exclude | FQ2 = No |
| 241 | DIGITAL TWIN TECHNOLOGY IN HEALTHCARE SYSTEM |  |  | Exclude | FQ2 = No |
| 242 | Challenges and directions for digital twin implementation in otorhinolaryngology | 2024 | 10.1007/s00405-024-08662-5 | Exclude | FQ2 = No |
| 243 | Digital Twin for Neurology: An Introduction to a New Frontier in Healthcare | 2024 |  | Exclude | FQ2 = No |
| 244 | Exploring digital twin technologies to examine transformation in healthcare systems | 2024 |  | Exclude | FQ2 = No |
| 245 | Digital twin empowered wireless healthcare monitoring for smart home | 2023 |  | Exclude | FQ2 = No |
| 246 | Leveraging Digital Twin Technology to Combat Cardiovascular Disease: A Comprehensive Review | 2024 |  | Exclude |  |
| 247 | Precision treatment platform enabled by whole body digital twin technology | 2023 |  | Exclude | FQ2 = No |
| 248 | Digital Twin, Didymos, Meets Digital Cousin, Didymium. From Paradox to Paradigm or a Paradoxical Paradigm? | 2024 |  | Exclude | Publication type is not match. |
| 249 | The medical digital twin assisted by reduced order models and mesh morphing | 2018 |  | Include |  |
| 250 | Edge-assisted human-to-virtual twin connectivity scheme for human digital twin frameworks | 2022 |  | Exclude | FQ1 = No |
| 251 | Smart Solution Using Digital Twin and IoT for Diabetic Retinopathy | 2023 |  | Exclude | FQ2 = No |
| 252 | From the visible human project to the digital twin | 2023 |  | Exclude | FQ2 = No |
