# Supplementary material for: Technologies, Clinical Applications, and Implementation Barriers of Digital Twins in Precision Cardiology: Systematic Review
Source: JMIR Cardio. 2026 Jan 8;10:e78499. doi: 10.2196/78499 (PMC12782626; doi:10.2196/78499)
Supplement: Multimedia Appendix 3 [file cardio-v10-e78499-s003.docx]

Multimedia Appendix 3. Data Extraction Form

| Category | Item (short title) | Description |
| --- | --- | --- |
| Article metadata | Article ID | Internal ID or code used to uniquely identify this article in the review database |
| Article metadata | Full citation | Complete bibliographic citation as it should appear in the reference list |
| Article metadata | Year of publication | Calendar year in which the article was published |
| Article metadata | Country / region | Country or geographical region where the study was conducted or data originated |
| Article metadata | Journal / conference | Name of the journal or conference where the work was published |
| Article metadata | Study type | Overall design of the study (e.g., modeling study, feasibility study, clinical validation, simulation-only) |
| Article metadata | Study population | Brief description of included patients/subjects and sample size, if applicable |
| Article metadata | Clinical setting | Context in which data were collected or model applied (e.g., hospital, registry, retrospective dataset, etc.) |
| Technological foundations | **RQ1 – Primary modeling approach** | Main modeling paradigm used for the digital twin as stated by the authors (mechanistic, data-driven, hybrid, etc.) |
| Technological foundations | **RQ2 – Mechanistic model type** | Type of mechanistic model if used (e.g., FEM, CFD, electromechanical, lumped parameter, other) |
| Technological foundations | **RQ3 – ML / AI algorithms used** | Machine learning or AI methods applied in building or personalizing the digital twin |
| Technological foundations | **RQ4 – Open-source availability** | Whether the framework/model is publicly available and, if so, how it can be accessed (e.g., URL, repository) |
| Data & visualization | **RQ5 – Patient-specific data types** | Types of patient-level data used to build, calibrate, or personalize the digital twin |
| Data & visualization | **RQ6 – Visualization format** | Main way that digital twin outputs are presented visually (e.g., 3D view, interface, figure, table, text) |
| Clinical applications | **RQ7 – Main clinical application** | Primary clinical purpose of the digital twin in this study (e.g., diagnosis, risk prediction, planning, monitoring) |
| Clinical applications | **RQ8 – Cardiovascular condition(s)** | Cardiovascular disease(s) or condition(s) targeted by the digital twin, using the terminology from the article |
| Clinical impact | **RQ9 – Reported clinical impact** | Any reported or claimed effect on clinical practice, decision-making, accuracy, or patient outcomes |
| Implementation barriers | **RQ10 – Practical / technical barriers** | Real-world technical or organizational obstacles to implementation or deployment in clinical workflows |
| Ethical & legal aspects | **RQ11 – Legal / ethical / governance issues** | Stated concerns about privacy, consent, regulation, fairness, or data governance related to the digital twin |
| General comments | Important quotations | Key sentences or passages that capture the essence of the study or its conclusions (with page/section reference) |
| General comments | Methodological strengths | Short summary of notable strengths or advantages of the study design or methods |
| General comments | Methodological limitations | Short summary of key limitations, biases, or weaknesses discussed or observed |
| Reviewer info | Reviewer initials | Initials or identifier of the reviewer who extracted the data |
| Reviewer info | Date of extraction | Date on which the data extraction for this article was completed |
