# Supplementary material for: Technologies, Clinical Applications, and Implementation Barriers of Digital Twins in Precision Cardiology: Systematic Review
Source: JMIR Cardio. 2026 Jan 8;10:e78499. doi: 10.2196/78499 (PMC12782626; doi:10.2196/78499)
Supplement: Checklist 1 [file cardio-v10-e78499-s006.pdf]

PRISMA 2020 Checklist

Section and Topic	Item #	Checklist item	Location where item is reported
**TITLE**			
Title	1	Identify the report as a systematic review.	Title page – manuscript title explicitly identifies the study as a systematic review.
**ABSTRACT**			
Abstract	2	See the PRISMA 2020 for Abstracts checklist.	Structured Abstract (Background, Objective, Methods, Results, Conclusions).
**INTRODUCTION**			
Rationale	3	Describe the rationale for the review in the context of existing knowledge.	Introduction – paragraphs describing cardiovascular disease burden, emerging role of digital twins in cardiology, current fragmentation of evidence, and need for a domain-specific synthesis.
Objectives	4	Provide an explicit statement of the objective(s) or question(s) the review addresses.	Introduction – paragraph beginning with the aim of the review ("To address this gap…") and the list of RQ1–RQ11, grouped into technological foundations, data infrastructure, clinical applications, clinical impact, implementation barriers, and ethical considerations.
**METHODS**			
Eligibility criteria	5	Specify the inclusion and exclusion criteria for the review and how studies were grouped for the syntheses.	Methods – "Eligibility Criteria" subsection (inclusion and exclusion bullets,

PRISMA 2020 Checklist

Section and Topic	Item #	Checklist item	Location where item is reported
			language restrictions, cardiovascular focus, digital twin definition, original research only) and statement that studies were not excluded based on study design; description that studies are grouped by the eleven RQs and six thematic domains.
Information sources	6	Specify all databases, registers, websites, organisations, reference lists and other sources searched or consulted to identify studies. Specify the date when each source was last searched or consulted.	Methods – “Data Sources and Search Strategy”: lists all databases (PubMed, Scopus, Web of Science, IEEE Xplore, Google Scholar), manual screening of reference lists, and the date range when each source was last searched.
Search strategy	7	Present the full search strategies for all databases, registers and websites, including any filters and limits used.	Methods – “Data Sources and Search Strategy” plus Multimedia Appendix 1 (full search strings and filters for each database).
Selection process	8	Specify the methods used to decide whether a study met the inclusion criteria of the review, including how many reviewers screened each record and each report retrieved, whether they worked independently, and if applicable, details of automation tools used in the process.	Methods – “Screening and Article Selection”: two-stage screening (title/abstract and full text), carried out independently by multiple reviewers with consensus resolution; description of use of shared Excel sheet; Figure 1 (PRISMA

PRISMA 2020 Checklist

Section and Topic	Item #	Checklist item	Location where item is reported
			2020 flow diagram); Multimedia Appendix 2 (full list of screened records with include/exclude status and reasons).
Data collection process	9	Specify the methods used to collect data from reports, including how many reviewers collected data from each report, whether they worked independently, any processes for obtaining or confirming data from study investigators, and if applicable, details of automation tools used in the process.	Methods – "Research Questions and Data Extraction" and "Data Management and Analysis": use of structured data extraction form in Excel, controlled vocabulary for coding, primary and secondary reviewer roles, and consensus resolution of disagreements; Multimedia Appendix 3 (completed extraction form for all included studies).
Data items	10a	List and define all outcomes for which data were sought. Specify whether all results that were compatible with each outcome domain in each study were sought (e.g. for all measures, time points, analyses), and if not, the methods used to decide which results to collect.	Methods – "Research Questions and Data Extraction" (RQ1–RQ11 define the main outcome domains: modeling approaches, mechanistic model types, ML/AI methods, openness, data modalities, visualization formats, clinical applications, conditions, clinical impact, limitations, legal/ethical issues); Methods – "Variables for Extraction" and Table 2 (summary of variables and thematic categories).
	10b	List and define all other variables for which data were sought (e.g. participant and intervention characteristics, funding sources). Describe	Methods – "Variables

PRISMA 2020 Checklist

Section and Topic	Item #	Checklist item	Location where item is reported
		any assumptions made about any missing or unclear information.	for Extraction" and Table 2: variables include study ID, reference, author(s), year, database source, journal/conference, study focus, digital twin definition, data sources, outcome measures, technology type, clinical condition, clinical application, and funding; Methods – "Data Management and Analysis" (description of coding and treatment of missing/not reported information). The full coded data extraction matrix is in Multimedia Appendix 3.
Study risk of bias assessment	11	Specify the methods used to assess risk of bias in the included studies, including details of the tool(s) used, how many reviewers assessed each study and whether they worked independently, and if applicable, details of automation tools used in the process.	Methods – "Risk of Bias Assessment": describes use of a Custom Modeling Checklist for 38 simulation/digital-twin modeling studies, PROBAST for 2 prediction-modeling studies, and ROBINS-I for 2 observational cohort studies; notes that two reviewers assessed risk of bias with consensus resolution. Supporting details and overall judgments are reported in Results – "Risk of Bias

PRISMA 2020 Checklist

Section and Topic	Item #	Checklist item	Location where item is reported
			Assessment," Table 3, Supplementary Figures (robvis plots)
Effect measures	12	Specify for each outcome the effect measure(s) (e.g. risk ratio, mean difference) used in the synthesis or presentation of results.	Methods – "Data Management and Analysis" (states that outcomes are synthesized descriptively using counts, proportions, and narrative summaries); Results – RQ1–RQ11 subsections (no standardized effect measures or meta-analytic estimates; no comparative effect sizes are calculated).
Synthesis methods	13a	Describe the processes used to decide which studies were eligible for each synthesis (e.g. tabulating the study intervention characteristics and comparing against the planned groups for each synthesis (item #5)).	Methods – "Research Questions and Data Extraction" and "Data Management and Analysis": each article could contribute to one or more of the eleven RQs; studies grouped into six thematic domains (technological foundations, data infrastructure, clinical applications, clinical impact, implementation challenges, ethics).
	13b	Describe any methods required to prepare the data for presentation or synthesis, such as handling of missing summary statistics, or data conversions.	Not applicable – no numerical data conversions or imputations were performed; all data were summarized as reported. This is stated in Methods –

PRISMA 2020 Checklist

Section and Topic	Item #	Checklist item	Location where item is reported
			"Data Management and Analysis."
	13c	Describe any methods used to tabulate or visually display results of individual studies and syntheses.	Methods – "Data Management and Analysis" (description of tabulation and thematic coding); Results – RQ1–RQ11 subsections and figures (e.g., summary tables, Sankey diagrams, and heatmaps); Multimedia Appendix 3 (full extraction matrix) and Multimedia Appendix 4 (study characteristics table).
	13d	Describe any methods used to synthesize results and provide a rationale for the choice(s). If meta-analysis was performed, describe the model(s), method(s) to identify the presence and extent of statistical heterogeneity, and software package(s) used.	Methods – "Data Management and Analysis" and opening of Results: explains that a narrative, thematic synthesis was performed structured by RQs and domains; no meta-analysis or statistical pooling was conducted.
	13e	Describe any methods used to explore possible causes of heterogeneity among study results (e.g. subgroup analysis, meta-regression).	Not applicable
	13f	Describe any sensitivity analyses conducted to assess robustness of the synthesized results.	Not applicable
Reporting bias assessment	14	Describe any methods used to assess risk of bias due to missing results in a synthesis (arising from reporting biases).	Not applicable – no formal assessment of reporting bias was conducted; this is noted in Methods – "Risk of Bias Assessment" and implicit in Discussion – "Limitations of This Review."
Certainty	15	Describe any methods used to assess certainty (or confidence) in the body of evidence for an outcome.	No formal

PRISMA 2020 Checklist

Section and Topic	Item #	Checklist item	Location where item is reported
assessment			certainty-of-evidence framework (such as GRADE) was applied. This is stated in Discussion – "Limitations of This Review," where the overall maturity and strength of the evidence are discussed narratively instead.
**RESULTS**			
Study selection	16a	Describe the results of the search and selection process, from the number of records identified in the search to the number of studies included in the review, ideally using a flow diagram.	Results – opening paragraph of "Study Selection / Results" and Methods – "Screening and Article Selection"; Figure 1 (PRISMA 2020 flow diagram showing 271 records identified and 42 studies included); Multimedia Appendix 2 (full list of records with inclusion/exclusion).
	16b	Cite studies that might appear to meet the inclusion criteria, but which were excluded, and explain why they were excluded.	Figure 1 PRISMA flow diagram (boxes indicating numbers and main reasons for exclusion, e.g., non-cardiac focus, not personalized, not original research, non-English, no full text) and Multimedia Appendix 2 (record-level information with include/exclude status and reasons where applicable).
Study characteristics	17	Cite each included study and present its characteristics.	Results – thematic subsections

PRISMA 2020 Checklist

Section and Topic	Item #	Checklist item	Location where item is reported
			(Technological Foundations, Data Infrastructure and Visualization, Clinical Applications and Conditions, Clinical Impact, Implementation Challenges, Ethical Considerations) where representative studies are cited; Multimedia Appendix 4 (detailed study characteristics table for all 42 studies, including design, population/condition, DT implementation, data inputs, clinical application, outcomes/impacts, funding, and overall risk-of-bias judgment); Multimedia Appendix 3 (completed extraction form).
Risk of bias in studies	18	Present assessments of risk of bias for each included study.	"Risk of Bias Assessment" subsection; Table 3 (summary of overall risk-of-bias judgments across tools); Supplementary Figures (robvis traffic-light and summary plots for modeling, prediction-modeling, and observational studies); per-study overall RoB judgments also summarized in Multimedia Appendix

Section and Topic	Item #	Checklist item	Location where item is reported
			4.
Results of individual studies	19	For all outcomes, present, for each study: (a) summary statistics for each group (where appropriate) and (b) an effect estimate and its precision (e.g. confidence/credible interval), ideally using structured tables or plots.	Results – RQ1–RQ11 subsections: frequencies and proportions for key categories (e.g., modeling approaches, mechanistic model types, ML families, data modalities, clinical applications and conditions, clinical impacts, limitations, ethical issues) and narrative summaries with representative study citations; detailed per-study coding is available in Multimedia Appendix 3 and Multimedia Appendix 4.
Results of syntheses	20a	For each synthesis, briefly summarise the characteristics and risk of bias among contributing studies.	Results – RQ1–RQ11 subsections and “Risk of Bias Assessment”; Discussion – “Sources and implications of heterogeneity” and “Limitations of This Review” (summarizes common risk-of-bias patterns and methodological limitations across modeling, prediction, and observational studies); Table 3 and Multimedia Appendix 4 (study-level characteristics and

PRISMA

PRISMA 2020 Checklist

Section and Topic	Item #	Checklist item	Location where item is reported
			RoB).
	20b	Present results of all statistical syntheses conducted. If meta-analysis was done, present for each the summary estimate and its precision (e.g. confidence/credible interval) and measures of statistical heterogeneity. If comparing groups, describe the direction of the effect.	Not applicable
	20c	Present results of all investigations of possible causes of heterogeneity among study results.	Not applicable
	20d	Present results of all sensitivity analyses conducted to assess the robustness of the synthesized results.	Not applicable
Reporting biases	21	Present assessments of risk of bias due to missing results (arising from reporting biases) for each synthesis assessed.	Not applicable
Certainty of evidence	22	Present assessments of certainty (or confidence) in the body of evidence for each outcome assessed.	Not applicable
**DISCUSSION**			
Discussion	23a	Provide a general interpretation of the results in the context of other evidence.	Discussion – opening paragraphs (“Technological Foundations and Modeling Strategies,” “Data Infrastructure and Visualization,” “Clinical Applications and Target Conditions,” “Impact on Clinical Practice,” “Barriers to Implementation and Ethical Considerations”).
	23b	Discuss any limitations of the evidence included in the review.	Discussion – “Limitations of This Review” (limitations of the included studies, such as small samples, limited validation, heterogeneity, incomplete reporting).
	23c	Discuss any limitations of the review processes used.	Discussion – “Limitations of This Review” (no protocol registration, database and language restrictions, lack of grey-literature and clinical-trial registry searches, heterogeneity

PRISMA

PRISMA 2020 Checklist

Section and Topic	Item #	Checklist item	Location where item is reported
			precluding meta-analysis, judgment-based adaptation of risk-of-bias tools).
	23d	Discuss implications of the results for practice, policy, and future research.	Discussion – “Implications and Future Directions” and “Conclusions” (implications for clinical adoption, standardization, validation, regulatory and ethical considerations, and directions for future work).
**OTHER INFORMATION**			
Registration and protocol	24a	Provide registration information for the review, including register name and registration number, or state that the review was not registered.	Discussion – “Limitations of This Review”: explicitly states that the review protocol was not registered on a public platform (e.g., OSF or PROSPERO).
	24b	Indicate where the review protocol can be accessed, or state that a protocol was not prepared.	Discussion – “Limitations of This Review”: notes that no formal protocol was prepared for registration.
	24c	Describe and explain any amendments to information provided at registration or in the protocol.	Not applicable
Support	25	Describe sources of financial or non-financial support for the review, and the role of the funders or sponsors in the review.	“Funding” statement: describes the grant(s) supporting the review (e.g., National Science Foundation award 2218046) and notes that funders had no role in study design, data collection, analysis, interpretation, or

PRISMA 2020 Checklist

Section and Topic	Item #	Checklist item	Location where item is reported
			manuscript preparation.
Competing interests	26	Declare any competing interests of review authors.	“Conflict of Interest” section: states that all authors declare no conflicts of interest relevant to this work.
Availability of data, code and other materials	27	Report which of the following are publicly available and where they can be found: template data collection forms; data extracted from included studies; data used for all analyses; analytic code; any other materials used in the review.	“Data Availability” section: states that all data were extracted from publicly available published studies; that the full list of screened records and their inclusion/exclusion status is provided in Multimedia Appendix 2; that the completed data extraction form is provided in Multimedia Appendix 3; that detailed study characteristics, RQ categories, risk-of-bias judgments, and funding information are provided in Multimedia Appendix 4; and that no analytic code or individual-level patient data were generated.

From: Page MJ, McKenzie JE, Bossuyt PM, Boutron I, Hoffmann TC, Mulrow CD, et al. The PRISMA 2020 statement: an updated guideline for reporting systematic reviews. BMJ 2021;372:n71. doi: 10.1136/bmj.n71. This work is licensed under CC BY 4.0. To view a copy of this license, visit https://creativecommons.org/licenses/by/4.0/
